# Identification and Validation of the Nrf2/Slc2a1 Axis in Alzheimer’s Disease

**DOI:** 10.3390/genes17091081

**Published:** 2026-09-08

**Authors:** Yantong Luo, Jingjing Deng, Yuanzhan Loh, Hongduo Lu, Nanbu Wang, Chunzhi Tang, Bin Zhang

**Affiliations:** 1 The First Clinical Medical College, Guangzhou University of Chinese Medicine, Guangzhou 510405, China; 2 Lingnan Medical Research Center, Guangzhou University of Chinese Medicine, Guangzhou 510405, China; 3 The Third Clinical Medical College, Guangzhou University of Chinese Medicine, Guangzhou 510405, China; 4 Medical College of Acu-Moxi and Rehabilitation, Guangzhou University of Chinese Medicine, Guangzhou 510405, China

**Keywords:** Alzheimer’s disease, oxidative stress, Nrf2, Slc2a1

## Abstract

Background/Objects: Alzheimer’s disease (AD) is a chronic neurodegenerative disorder characterized by progressive cognitive decline. Although Nrf2 is a key regulator of oxidative stress in AD, the molecular factors associated with Nrf2 signaling and their potential regulatory relationships remain incompletely understood. This study aimed to systematically identify and validate a novel Nrf2-associated signaling axis in AD. Results: Slc2a1 was identified as a key Nrf2-associated candidate gene. In the SCOP-induced AD model, reduced Nrf2 expression was accompanied by decreased Slc2a1 expression, cognitive impairment, hippocampal neuronal injury, excessive ROS accumulation, and mitochondrial abnormalities. Pharmacological modulation of Nrf2 was associated with corresponding changes in Slc2a1 expression, while Nrf2 activation was accompanied by improvements in oxidative stress and neuronal injury. Conclusions: This study identifies a potential functional relationship between Nrf2 signaling and Slc2a1 expression in AD-related pathology and proposes the Nrf2/Slc2a1 pathway as a potential mechanistic framework linking oxidative stress to mitochondrial abnormalities.

## 1. Introduction

Alzheimer’s disease (AD) is a debilitating neurodegenerative condition marked by progressive cognitive impairment and widespread neuronal degeneration [1]. With the rapid aging of the global population, the prevalence of AD continues to rise, posing an increasing burden on patients, caregivers, and healthcare systems worldwide [2]. Current pharmacological therapies primarily provide symptomatic relief and only modest, transient improvements in cognitive function, without effectively halting disease progression [3]. Consequently, deciphering the biological processes driving AD progression and uncovering new opportunities for therapeutic intervention remain pressing priorities in AD research.

Oxidative stress is a key driver of AD pathogenesis [4]. As the principal regulator of the antioxidant defense network, Nrf2 is crucial for maintaining redox homeostasis and neuronal survival [5]. Impaired Nrf2 expression or activity has been consistently associated with excessive ROS accumulation, neuronal injury, and cognitive impairment in both patients with AD and experimental models [6]. However, existing studies have largely concentrated on validating the canonical target genes of Nrf2, such as heme oxygenase-1 (HO-1) and NAD(P)H quinone oxidoreductase 1 (NQO1), which primarily mediate antioxidant defense [7]. In contrast, whether additional Nrf2-associated molecular factors participate in the neuroprotective actions of Nrf2 during AD progression remains largely unexplored. This knowledge gap has hindered a comprehensive understanding of the Nrf2 regulatory network in AD. Traditional candidate gene approaches are limited by prior knowledge bias, whereas integrating transcriptomic profiling with multiple machine learning algorithms allows unbiased identification of key hub genes from high-dimensional omics data, significantly reducing false positive rates and improving the reliability of target discovery.

The 8-week-old male C57BL/6 mouse was selected as the experimental animal for this study due to its stable genetic background and wide application in neuroscience research, which ensures the reproducibility and translational relevance of experimental results. The scopolamine (SCOP)-induced AD model was chosen because it can specifically mimic the cholinergic system damage, oxidative stress imbalance, and cognitive dysfunction that are core pathological features of early AD, and the modeling method is mature, controllable, and highly reproducible, making it suitable for studying the molecular mechanisms of oxidative stress-related neuronal injury in AD. This study first clarified the important roles of oxidative stress and Nrf2 in AD. Subsequently, peripheral whole-blood transcriptomic profiling was employed as an initial discovery strategy to identify systemic molecular alterations and candidate genes associated with Nrf2 in SCOP-treated mice. Peripheral blood was selected as an accessible source for candidate-gene discovery rather than as a surrogate for hippocampal gene expression. Given the tissue-specific nature of transcriptomic profiles, peripheral blood transcriptomic patterns may be influenced by circulating cell composition and systemic responses. Therefore, we further investigated the identified candidate genes in hippocampal tissue and HT22 cells to assess their potential association with pathological alterations in the central nervous system. In addition, we preliminarily investigated the potential relationship among oxidative stress, Nrf2-associated candidate genes, and mitochondrial abnormalities. The valuable insights obtained from these findings have significantly enhanced our understanding of the complex oxidative stress mechanisms associated with AD.

### Objectives

The overall objective of this study is to systematically identify and validate novel Nrf2-associated genes in AD, and to explore their potential roles in oxidative stress-induced neuronal injury. Specifically, this study aims to test the following core scientific hypothesis: We hypothesized that Nrf2 signaling is functionally associated with Slc2a1 expression and that the Nrf2/Slc2a1 pathway may be involved in oxidative stress and mitochondrial abnormalities in AD-related pathological conditions. To address this hypothesis, we pursue three specific aims: (1) To initially screen Nrf2-associated candidate genes using peripheral blood transcriptomic profiling and multi-algorithm machine learning in the SCOP-induced AD mouse model. (2) To further investigate the association between Nrf2 and the identified candidate gene Slc2a1 in hippocampal tissue and HT22 cells. (3) To explore the potential relationship between Slc2a1 and mitochondrial abnormalities under oxidative stress conditions.

## 2. Materials and Methods

### 2.1. Animal Experiments

**Figure 1:**
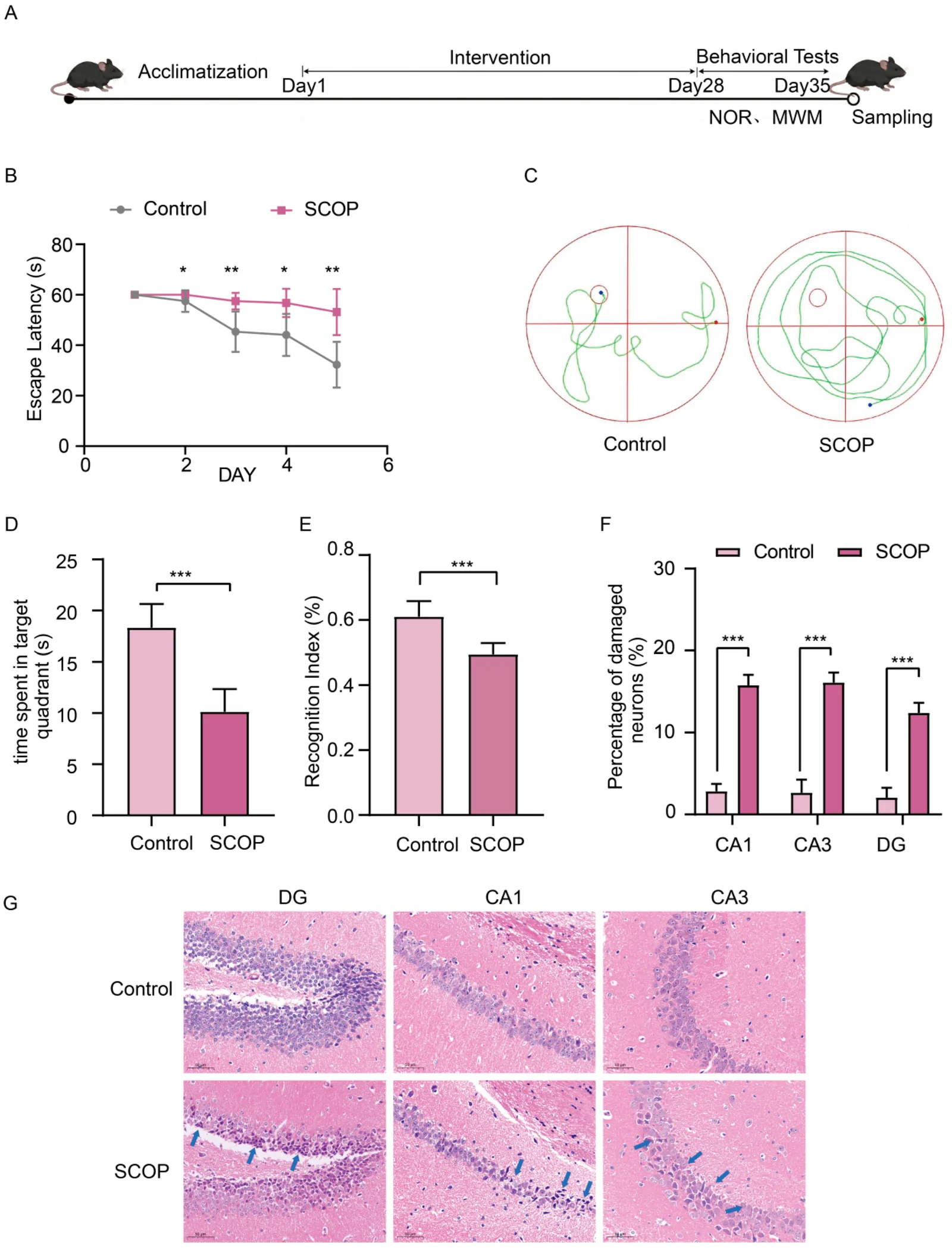
Impact of SCOP Treatment on Cognitive Function and Hippocampal Neuronal Loss in C57 Mice. (**A**) Experimental schedule and workflow. (**B**) MWM acquisition escape latency in Control and SCOP groups (*n* = 10). (**C**) Day 5 representative MWM swimming paths. (**D**) Target quadrant duration during MWM probe trial. (**E**) NOR discrimination index. (**F**) Quantification of surviving neurons across hippocampal DG, CA1, and CA3 subfields from HE staining. (**G**) Representative HE images of DG, CA1, and CA3 regions (magnification: 50×, scale bar: 50 μm). Abnormal neurons are indicated by blue arrows. (* *p* < 0.05, ** *p* < 0.01, *** *p* < 0.001. SCOP: scopolamine).

Fifty 8-week-old male C57BL/6 mice were selected for this study. All animals were housed in a specific pathogen-free (SPF) environment with ad libitum access to standard chow and water. Following a 1-week acclimatization period, mice with subnormal body weight were excluded. The remaining animals were randomly divided into 5 primary groups (10 mice per group): (1) Blank Control, (2) Scopolamine model (SCOP), (3) SCOP + XJB-5-131 intervention, (4) SCOP + TBHQ intervention, alongside a separate Phloretin administration group. To establish an animal model of Alzheimer’s disease-like cognitive impairment, mice in the SCOP group received daily intraperitoneal (i.p.) injections of 5 mg/kg scopolamine (Cat# HY-N0296, MedChemExpress [MCE], Monmouth Junction, NJ, USA). On the basis of model induction, the SCOP + XJB group was additionally administered 2 mg/kg XJB-5-131 (a reactive oxygen species scavenger; Cat# HY-129460, MCE) via i.p. injection three times a week. The SCOP + TBHQ group received daily i.p. injections of 5 mg/kg TBHQ (an Nrf2 activator; Cat# HY-100489, MCE). The Blank Control group received an equal volume of normal saline via i.p. injection daily. The Phloretin group received daily i.p. injections of 10 mg/kg phloretin (an Slc2a1 inhibitor) alone. All corresponding interventions were carried out continuously for 30 days. All animal handling and experimental procedures strictly complied with the guidelines approved by the Animal Ethics Committee of Guangzhou University of Chinese Medicine (Approval No. 20241225005) (Figure 1A). All animals were humanely euthanized at the experimental endpoint by cervical dislocation under deep isoflurane anesthesia, ensuring that the animals were free from pain and distress throughout the entire procedure.

### 2.2. Morris Water Maze Test

The MWM test was used to assess spatial learning and memory in mice. The test was performed in a circular tank (diameter: 120 cm, height: 50 cm) split into four equal quadrants. A target platform (6 cm diameter) was submerged 1.5 cm below opaque, TiO_2_-treated water (23 ± 2 °C). Acquisition training lasted 5 days. Daily trials started from four distinct wall-facing locations. Mice were allowed 60 s to find the platform. Upon arrival, a 15 s rest was granted. Guided placement (15 s) was provided for failed trials. Latencies and trajectories were collected. A probe test was executed on day 6. The platform was removed. Animals were placed in the opposite quadrant for a 60 s free swim. Target quadrant time was calculated. Testing occurred during the active phase (19:00). Data were analyzed via a video-tracking software (Shanghai Jiliang Software Technology Co., Ltd., Shanghai, China).

### 2.3. Novel Object Recognition Test

The Novel Object Recognition (NOR) test was conducted to assess recognition memory in mice. Cognitive function was measured via the NOR task inside an opaque open field (40 × 40 × 40 cm). Environmental habituation was performed on day 1 by placing single mice in the empty arena for 10 min. The training trial was conducted 24 h later using two symmetrical identical objects for 10 min. Following a 24-h interval, one familiar object was swapped for a novel object during a 5-min test phase. Object-directed activity (nose distance < 2cm or physical contact) was logged using a computerized tracking system (Jiliang Software, Shanghai, China). Preference index was determined via PI (%) = Tn/Tf + Tn. Olfactory cues were eliminated by wiping all surfaces with 75% ethanol after each trial.

### 2.4. Omics Profiling and Bioinformatic Analysis

Peripheral blood transcriptomic profiling was performed as an initial discovery approach to identify systemic molecular alterations and screen for candidate Nrf2-associated genes in SCOP-treated mice. Peripheral blood was collected from Control and SCOP mice (*n* = 3 per group, with each sample derived from an individual animal). The transcriptomic findings were used for candidate-gene discovery. Total RNA was extracted and quality-checked. Libraries were constructed and sequenced via high-throughput platforms. Low-quality reads and adapters were filtered. DEGs were identified using |logFC| > 1.0 and a raw *p* value < 0.05 as the screening criteria. Multiple-testing correction was performed using the Benjamini–Hochberg method to calculate FDR-adjusted *p* values [8]. GO and KEGG functional enrichments were performed via “clusterProfiler” [9]. Nrf2-related DEGs were obtained by intersecting DEGs with a curated Nrf2 geneset. Feature selection was conducted using LASSO regression, SVM-RFE, and Random Forest algorithms to identify key disease-related hub genes [10,11,12]. Candidate gene expressions were compared between Control and SCOP groups.

### 2.5. HE Staining

HE staining was performed to evaluate histopathological alterations in the hippocampus. Brain specimens were fixed (4% paraformaldehyde, Biosharp, BL539A, >48 h) and trimmed. Dehydration was conducted through graded ethanol, followed by clearing in alcohol–benzene and xylene. Tissues were then infiltrated and embedded in paraffin. Microtome coronal sectioning (4 μm) was performed. Sections were mounted, dried, deparaffinized, and rehydrated. Nuclear staining was executed with hematoxylin (P0148-1, Pinuofei Biotechnology, Wuhan, China), followed by differentiation (Pinuofei, P0148-3) and bluing (Pinuofei, P0148-4). Cytoplasm was counterstained with eosin (Pinuofei, P0148-2). Slides were re-dehydrated, cleared in xylene, and coverslipped using neutral medium. Histological images were acquired using a NIKON ECLIPSE Ci microscope (Nikon, Tokyo, Japan).

### 2.6. Immunofluorescence Staining

Immunofluorescence staining was performed to evaluate the expression and distribution of target proteins in hippocampal tissues. Five random specimens per group were selected. Sections were deparaffinized and rehydrated via xylene (10023418, Sinopharm, Shanghai, China) and graded ethanol (Sinopharm, 10009228). Antigen retrieval was performed using Tris-EDTA buffer (Pinofi, P0027). Endogenous peroxidase was quenched with 3% H_2_O_2_, followed by blocking with 10% goat serum (Pinofi, PN0038). Sections were incubated with the first primary antibody overnight at 4 °C, then secondary antibody (Pinofi, PN0046, 1 h, 37 °C). Signals were developed using TYR-488 TSA reagent (Pinofi, PN0100, 30 min). After a second antigen retrieval, the procedure was repeated with the second primary antibody and TYR-555 TSA (Pinofi, PN0101). Nuclei were counterstained with DAPI (Pinofi, PN0015). Sections were PBS-washed (Pinofi, PN0031), mounted with antifade medium (Pinofi, PN0024), and stored at 4 °C in the dark.

### 2.7. ROS Staining

Intracellular ROS levels were assessed using dihydroethidium (DHE) (S0063, Beyotime Biotechnology, Shanghai, China) staining. Briefly, frozen tissue sections were incubated with DHE working solution (PBS:DHE = 200:1) at 37 °C for 30 min in the absence of light. After PBS washes, nuclei were counterstained with DAPI, and the sections were mounted using an antifade mounting medium. Fluorescence signals were visualized and imaged using a Nikon inverted fluorescence microscope.

### 2.8. Western Blot

Western blotting was performed to quantify the expression levels of target proteins and evaluate alterations in the corresponding molecular pathways. Protein samples were resolved by 10% sodium dodecyl sulfate–polyacrylamide gel electrophoresis (SDS-PAGE) and subsequently electrotransferred to polyvinylidene fluoride (PVDF) membranes. After blocking for 15 min at room temperature, the membranes were incubated with primary antibodies overnight at 4 °C. On the subsequent day, membranes were exposed to HRP-conjugated secondary antibodies (WLA023, Wanlei, Shenyang, China), and immunoreactive bands were detected using an electrochemiluminescence-based infrared imaging system.

### 2.9. Transmission Electron Microscopy (TEM) Observation

TEM was used to examine mitochondrial ultrastructural changes in hippocampal tissues. Hippocampal tissues were excised (1–3 mm^3^) and fixed in EM fixative at 4 °C. Samples were post-fixed in 1% osmium tetroxide, dehydrated using graded ethanol and acetone, and embedded in 812 epoxy resin. Ultrathin sections (60–80 nm) were cut with an ultramicrotome and loaded onto copper grids. Sections were stained with uranyl acetate and lead citrate. Ultrastructural imaging was performed using a transmission electron microscope (HT7800, Hitachi, Tokyo, Japan).

### 2.10. Cell Proliferation Assay

The CCK-8 assay was performed to evaluate the effects of different treatment conditions on HT22 cell viability and to determine appropriate treatment concentrations for subsequent experiments. HT22 cells were generously provided by Dr. Xiaofang Guo [13]. Following the indicated treatments, the culture medium was aspirated and the cells were gently rinsed with PBS. Subsequently, 100 μL of fresh culture medium supplemented with 10 μL of Cell Counting Kit-8 (CCK-8) reagent (Elabscience Biotechnology Co., Ltd., Wuhan, China [E-CK-A362]) was added to each well, while blank wells containing medium alone served as controls. After incubation at 37 °C in a humidified atmosphere containing 5% CO_2_ for 2 h, absorbance was measured at 450 nm using a Multiskan GO microplate spectrophotometer (Model 1510; Thermo Fisher Scientific Oy, Vantaa, Finland) to assess cell viability.

### 2.11. Statistical Analysis

Bioinformatic workflows were executed in R (v4.3.1), and statistical tests were run using SPSS (v24.0). Multi-group data were analyzed using one-way ANOVA. Two-group data were assessed using either an unpaired Student’s t-test or a Mann–Whitney U test according to data variance. Statistical significance was set at *p* < 0.05.

## 3. Results

### 3.1. SCOP Triggers Cognitive Dysfunction and Hippocampal Neuronal Degeneration in Mice

To validate the SCOP-induced AD mouse model, cognitive behaviors and hippocampal histopathological changes were assessed via MWM, NOR tests, and HE staining. In the MWM acquisition phase, both groups showed decreased escape latency over time, but SCOP-treated mice exhibited significantly longer latencies than controls (Figure 1B). Day 5 swimming trajectories revealed disorganized navigation in the SCOP group, contrasted with the direct, goal-oriented paths of control mice (Figure 1C), indicating impaired spatial learning. Spatial memory retention was compromised in SCOP mice, as demonstrated by decreased occupancy in the target quadrant during probe testing (Figure 1D). Deficits in non-spatial recognition memory were likewise confirmed by reduced novel object preference in the NOR assay (Figure 1E). In control brains, intact, aligned neuronal architecture was displayed in hippocampal DG, CA1, and CA3 areas via HE staining. Conversely, SCOP treatment caused distinct histological damage, including disordered neuronal arrangement, decreased density, and cell shrinkage. Quantitative assessment verified a marked loss of surviving hippocampal neurons in the SCOP group (Figure 1F,G). Together, these behavioral and histological outcomes provide definitive evidence of significant cognitive impairment and neuronal injury in the AD model mice.

### 3.2. Oxidative Stress Is a Prominent Pathological Feature in SCOP-Induced AD Mice

**Figure 2:**
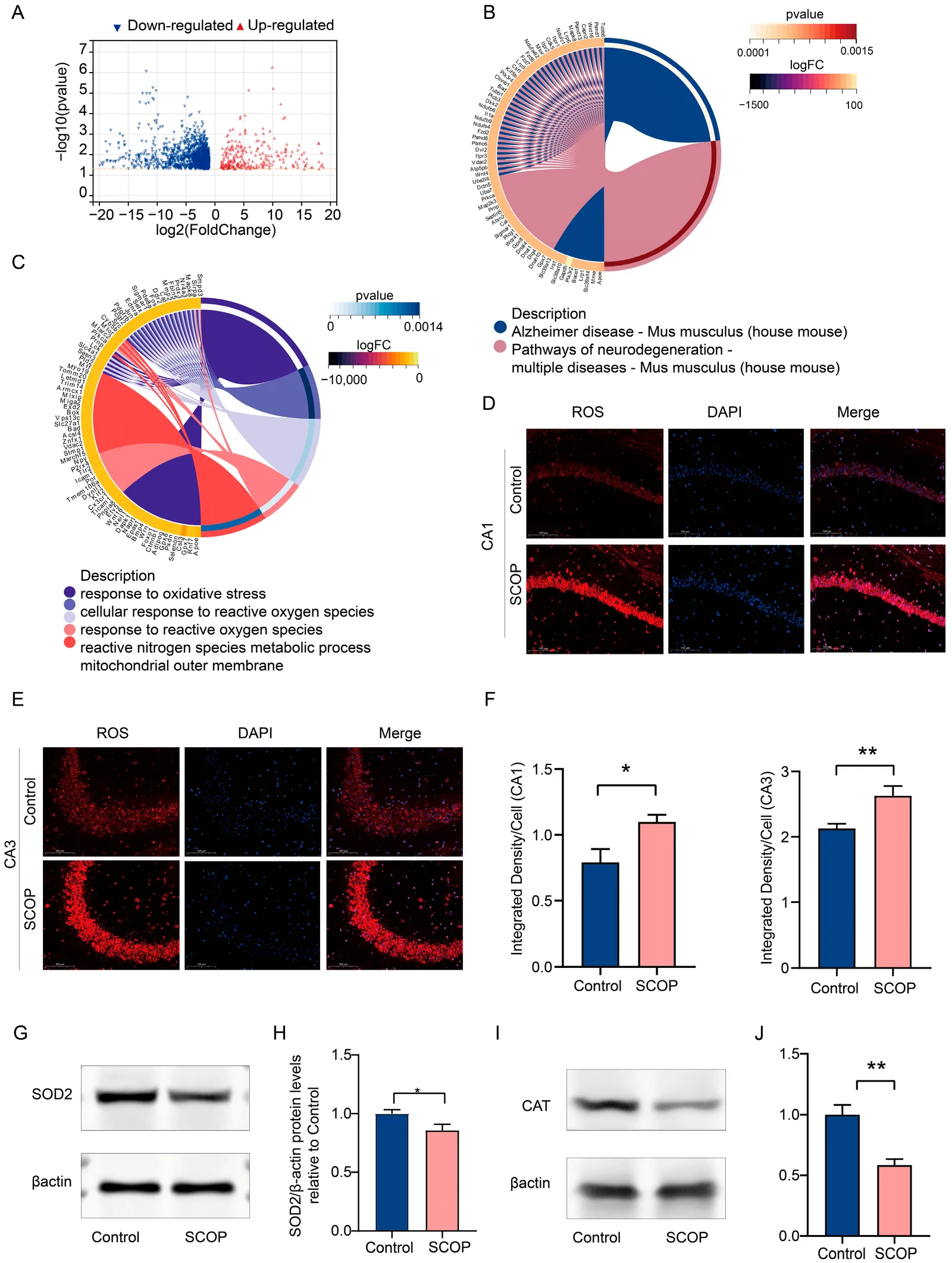
Transcriptomic Profiling of SCOP-Induced AD Mice and Its Association with Oxidative Stress. (**A**) Volcano plot visualization of DEGs derived from Control vs. SCOP group comparisons. Upregulated genes are marked by red dots, whereas downregulated genes are denoted by blue dots. (**B**) KEGG pathway enrichment mapping of DEGs. (**C**) GO functional annotation profiling of DEGs. (**D**,**E**) Microscopic immunofluorescence evaluation of ROS staining (red) within hippocampal CA1 and CA3 subfields from Control and SCOP animals (magnification, 40×; scale bar = 100 μm). (**F**) Densitometric evaluation of ROS fluorescent intensity across hippocampal slices. (**G**) Western blot bands depicting hippocampal SOD2 protein abundance. (**H**) Relative protein expression of SOD2 determined by densitometric analysis. (**I**) Western blot bands depicting hippocampal CAT protein abundance. (**J**) Relative protein expression of CAT determined by densitometric analysis. (* *p* < 0.05, ** *p* < 0.01, SCOP, scopolamine-treated AD mice).

To identify systemic molecular alterations associated with the SCOP-induced pathological state and generate candidate molecular signatures for subsequent investigation, peripheral blood samples from the Control and SCOP groups were subjected to RNA-seq analysis. Through differential expression screening, a total of 1744 DEGs were identified, among which 308 transcripts were upregulated and 1436 transcripts were downregulated in SCOP-treated animals compared to Control mice (Figure 2A, Table S1). Functional characterization was subsequently performed using KEGG pathway enrichment and GO annotation systems. The KEGG pathway analysis revealed that these DEGs were significantly involved in human neurodegenerative conditions, particularly Alzheimer’s disease pathways (Figure 2B). Furthermore, GO annotation demonstrated that dysregulated transcripts were heavily concentrated in biological processes associated with oxidative stress and redox imbalance, including cellular responses to ROS, nitrogen species metabolic activities, and functions related to the mitochondrial outer membrane (Figure 2C). To directly verify the presence of in vivo oxidative stress within brain tissue, immunofluorescence staining targeting ROS was conducted on hippocampal sections. A marked elevation in ROS fluorescence intensity was observed within both the CA1 and CA3 subfields of the SCOP group relative to the Control group (Figure 2D–F), confirming substantial oxidative overload. In agreement with these observations, Western blot assays revealed that the protein expression levels of key endogenous antioxidant enzymes, specifically SOD2 and CAT, were significantly suppressed in the hippocampal tissues of SCOP mice (Figure 2G–J). Taken together, peripheral blood transcriptomic analysis revealed systemic molecular alterations associated with oxidative stress, while subsequent hippocampal analyses independently confirmed marked oxidative stress in SCOP-treated mice, as evidenced by increased ROS accumulation and reduced SOD2 and CAT expression.

### 3.3. Nrf2 Suppression Is Associated with Cognitive Impairment and Hippocampal Injury Under Oxidative Stress

**Figure 3:**
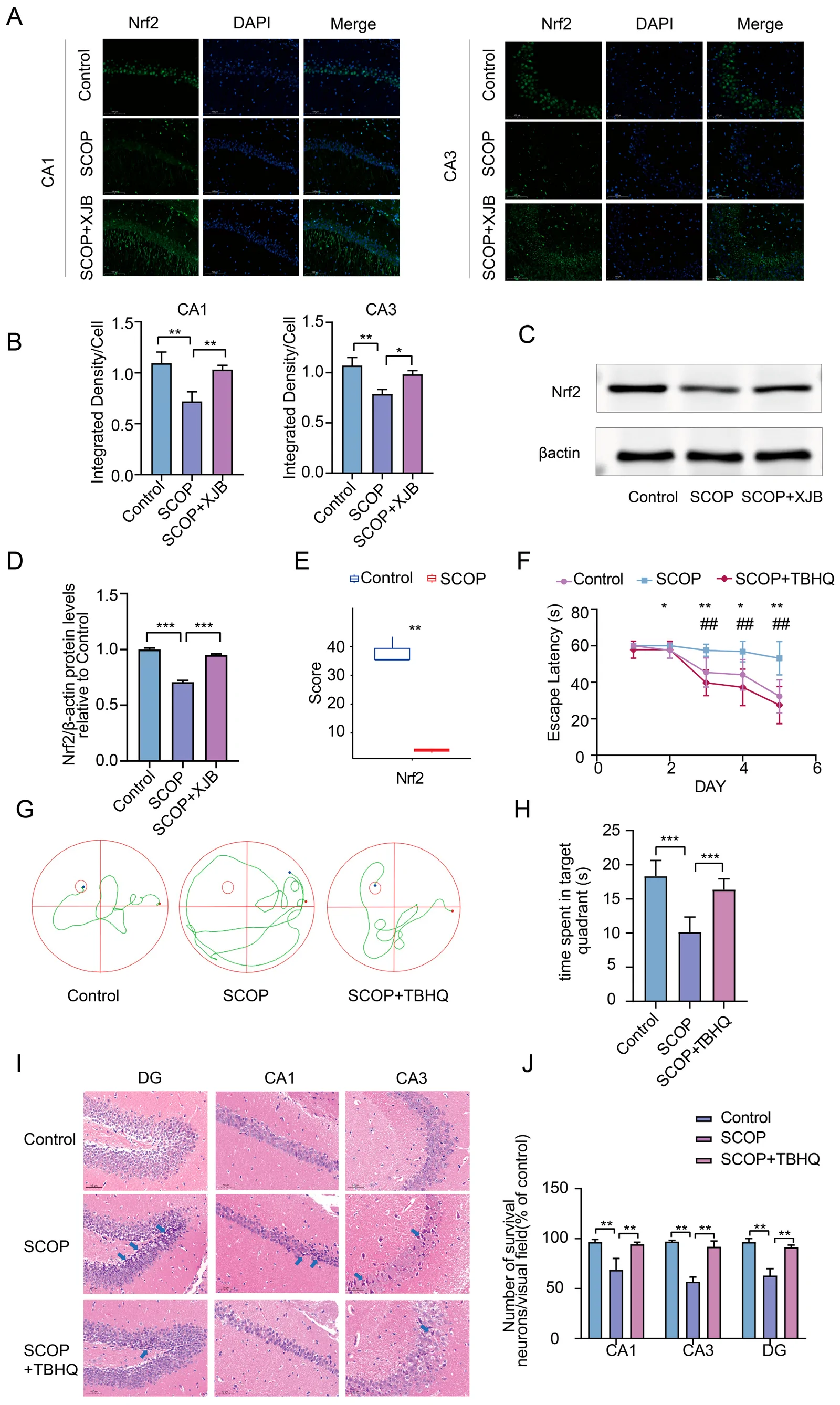
Activation of the Nrf2 Pathway Mitigates Oxidative Damage and Cognitive Impairment in AD Mice. (**A**) Confocal fluorescence micrographs displaying hippocampal Nrf2 levels in CA1 and CA3 subfields (magnification, 40×; scale bar = 100 μm). (**B**) Fluorescent intensity measurements of Nrf2 across treatment groups. (**C**) Immunoblot bands depicting Nrf2 protein levels in hippocampal tissue homogenates. (**D**) Relative expression of Nrf2 protein determined by densitometric scoring. (**E**) Boxplot showing Nrf2 transcript expression in peripheral blood RNA-seq profiles from the Control and SCOP groups. (**F**) Acquisition latency recorded during spatial learning trials in the Morris water maze (*n* = 10). (**G**) Swim path tracings recorded on training day 5. (**H**) Duration spent swimming within the target quadrant during the probe phase. (**I**) Histological micrographs of hippocampal subregions (CA1, CA3, and DG) stained with HE Abnormal neurons are indicated by blue arrows. (magnification, 50×; scale bar = 50 μm). (**J**) Survival neuron density evaluated in DG, CA1, and CA3 areas using HE-stained slices. (* *p* < 0.05, ** *p* < 0.01, *** *p* < 0.001 vs. the SCOP + TBHQ group; ^##^ *p* < 0.01 vs. the Control; SCOP, scopolamine-treated AD model; SCOP + XJB, scopolamine mice administered the ROS scavenger XJB-5-131 intraperitoneally; SCOP + TBHQ, scopolamine mice administered the Nrf2 activator TBHQ intraperitoneally).

As a key transcriptional regulator, Nrf2 orchestrates antioxidant defense mechanisms and represents a potential therapeutic avenue for Alzheimer’s disease treatment. To characterize the dynamic alterations of Nrf2 under conditions of oxidative stress, SCOP-treated mice were administered the ROS scavenger XJB-5-131 (SCOP + XJB group). Immunofluorescence staining (Figure 3A,B) and WB analysis (Figure 3C,D) consistently demonstrated a marked reduction in hippocampal Nrf2 expression following SCOP exposure, whereas XJB-5-131 treatment effectively restored Nrf2 levels. In parallel, peripheral blood RNA-seq analysis revealed reduced Nrf2 transcript abundance in SCOP-treated mice (Figure 3E). To further delineate the functional relevance of Nrf2 activation, mice were treated with the Nrf2 agonist TBHQ (SCOP + TBHQ group). In the MWM test, TBHQ administration significantly shortened escape latency beginning on day 3, and this improvement persisted throughout the subsequent training period (Figure 3F). Representative swimming trajectories recorded on day 5 demonstrated more efficient and goal-directed navigation in the SCOP + TBHQ group compared with the SCOP group (Figure 3G). Assessment of the probe phase revealed that TBHQ-treated mice showed greater preference for the target quadrant than SCOP mice (Figure 3H), suggesting enhanced spatial learning and memory retention after Nrf2 activation. The neuroprotective effects of Nrf2 activation were further substantiated by histopathological evaluation. HE staining revealed that TBHQ treatment largely preserved hippocampal cytoarchitecture, as evidenced by improved neuronal organization, increased cellular density within the DG, CA1, and CA3 regions, and a notable attenuation of neuronal shrinkage and necrotic alterations relative to SCOP-treated mice (Figure 3I). Quantitative assessment of surviving neurons corroborated these histological observations (Figure 3J), suggesting that pharmacological activation of Nrf2 effectively mitigates hippocampal neurodegeneration in this AD model. Collectively, these findings demonstrate an association between oxidative stress-related Nrf2 suppression, cognitive impairment, and hippocampal neuronal injury in SCOP-treated mice, while pharmacological activation of Nrf2 was accompanied by improvements in these pathological alterations.

### 3.4. Slc2a1 Is Identified as a Key Nrf2-Associated Hub Gene in AD

**Figure 4:**
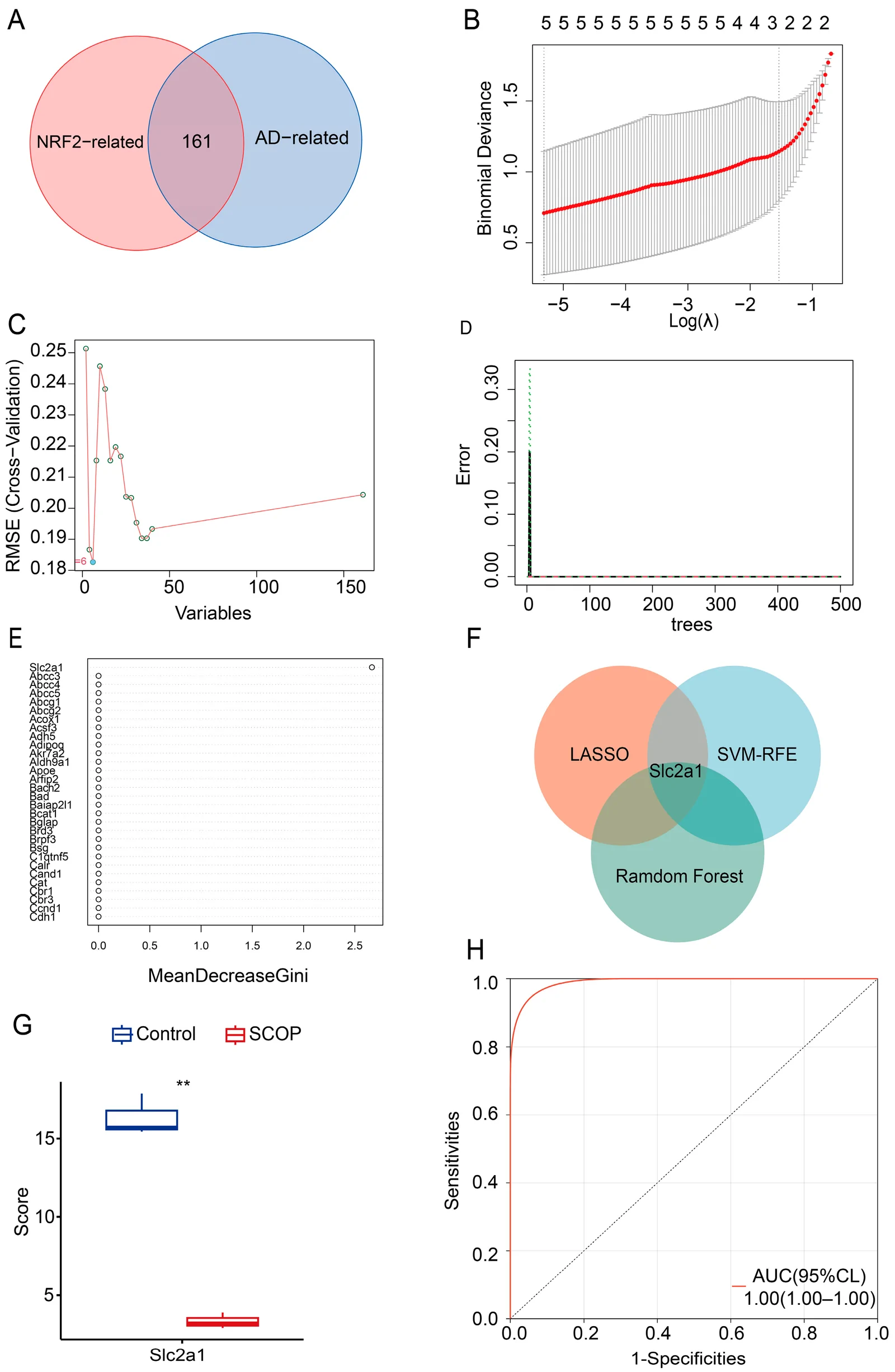
Identification of Nrf2-Associated Genes in AD Mice via RNA-Seq. (**A**) Venn diagram displaying the intersection of identified DEGs (blue) and curated Nrf2-related genes (red), from which 161 overlapping candidate genes were obtained. (**B**–**F**) Core gene selection executed via an integrative machine learning framework. Algorithms including LASSO regression (**B**), SVM-RFE (**C**), and Random Forest (**D**,**E**) were implemented on the 161 overlapping targets, culminating in the selection of Slc2a1 as a key hub candidate (**F**). (**G**) Relative expression levels of Slc2a1 compared between Control and SCOP groups. (**H**) Receiver operating characteristic (ROC) curve analysis of Slc2a1 constructed using the GSE122063 dataset to assess its performance as a potential diagnostic marker for AD. (** *p* < 0.01).

To further identify key molecular factors associated with Nrf2 signaling in AD, we conducted an integrative analysis incorporating both public datasets and our transcriptomic profiling data. A curated Nrf2-associated gene set was first retrieved from GeneCards and intersected with the DEGs identified from RNA-seq analysis, yielding 161 Nrf2-related DEGs (Figure 4A). To refine candidate selection, three complementary machine learning approaches, including LASSO regression (Figure 4B), SVM-RFE (Figure 4C), and random forest analysis (Figure 4D,E), were applied to the 161-gene dataset. Integration of the outputs from these independent algorithms converged on a single candidate, Slc2a1, as the putative hub gene (Figure 4F). Subsequent expression analysis demonstrated a significant reduction in Slc2a1 expression in SCOP-treated mice compared with Controls (Figure 4G), implicating its dysregulation in AD-associated pathological processes. To further assess its potential translational relevance, receiver operating characteristic (ROC) curve analysis was performed using the publicly available dataset GSE122063. Notably, Slc2a1 achieved an AUC value of 1.0 (Figure 4H), indicating outstanding discriminatory capacity and further supporting its potential significance in AD. Collectively, these findings identify Slc2a1 as a key Nrf2-associated hub gene in the SCOP-induced AD model.

### 3.5. Modulation of Nrf2 Activity Is Associated with Altered Slc2a1 Expression In Vitro and In Vivo

**Figure 5:**
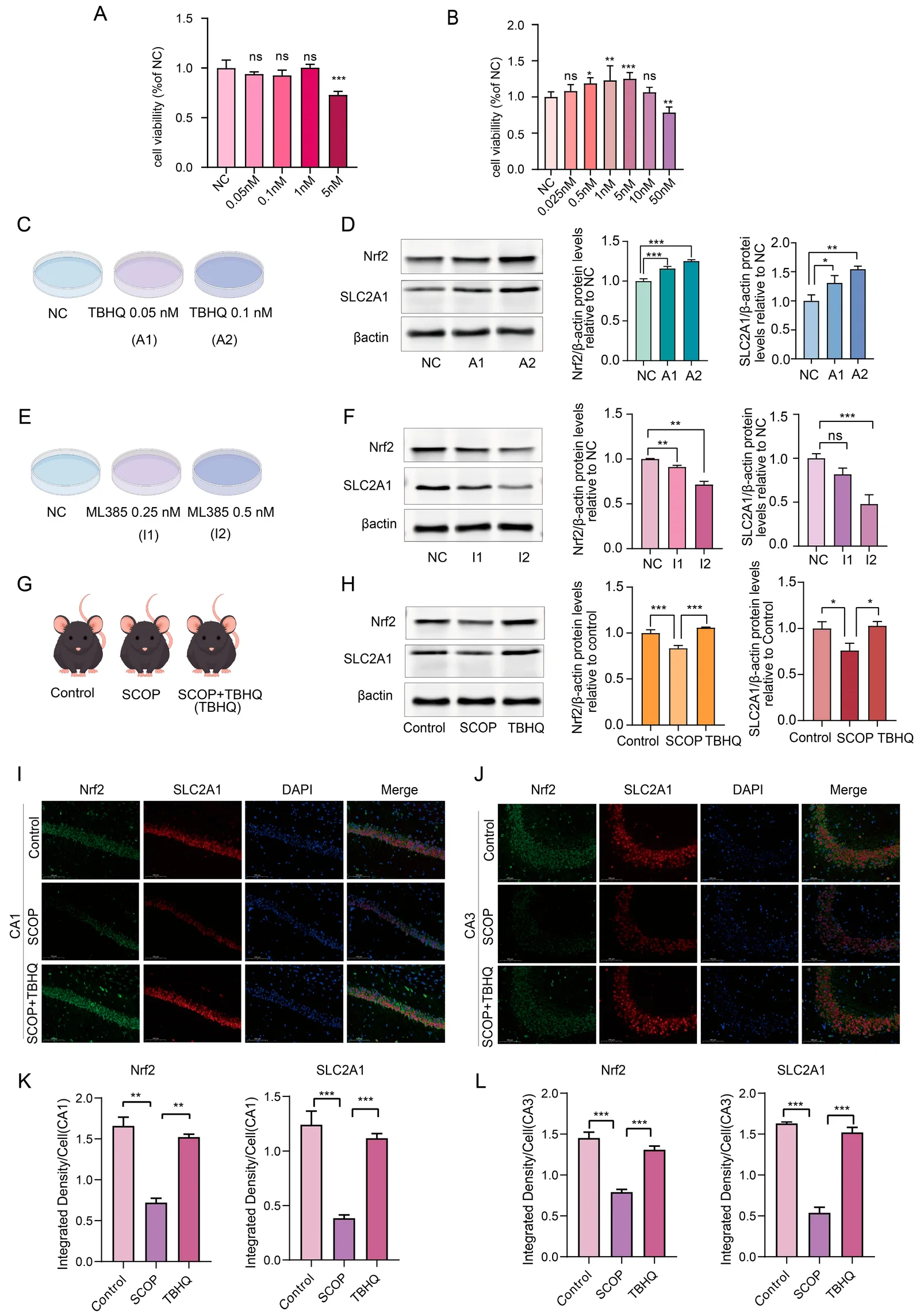
Pharmacological Modulation of Nrf2 Is Associated with Altered Slc2a1 Expression. (**A**,**B**) CCK-8 assays were performed to determine the concentration ranges of TBHQ (**A**) and ML385 (**B**) in HT22 cells. (**C**) Schematic illustration of the experimental design for TBHQ treatment in HT22 cells. (**D**) Representative WB images and quantification of Nrf2 and Slc2a1 protein expression in HT22 cells following TBHQ treatment. (**E**) Schematic illustration of the experimental design for ML385 treatment in HT22 cells. (**F**) Representative WB images and quantification of Nrf2 and Slc2a1 protein expression in HT22 cells following ML385 treatment. (**G**,**H**) Representative WB images of Nrf2 and Slc2a1 protein expression in hippocampal tissue (**G**) and corresponding analysis (**H**). (**I**,**J**) Representative immunofluorescence images showing Nrf2 (green) and Slc2a1 (red) expression in the CA1 and CA3 regions of the hippocampus (magnification, 40×; scale bar = 100 μm). (**K**,**L**) Quantitative analysis of Nrf2 and Slc2a1 immunofluorescence signal intensity. (Note: “*”, “**”, “***” represents *p* < 0.05, *p* < 0.01, *p* < 0.001; ns, not significant).

To elucidate the regulatory interplay between Nrf2 and Slc2a1, a series of complementary in vivo and in vitro investigations were conducted. The murine hippocampal neuronal cell line HT22 was employed as an experimental model to characterize the mechanistic relationship between these two molecules. Initially, HT22 cells were exposed to either the Nrf2 activator TBHQ and the selective Nrf2 inhibitor ML385 to modulate Nrf2 signaling activity. Prior to mechanistic analyses, a CCK-8 assay was conducted to evaluate the cytotoxicity of both compounds and establish appropriate treatment concentrations. The results indicated that neither TBHQ (0.05–1 nM; Figure 5A) nor ML385 (0.5–10 nM; Figure 5B) exerted significant effects on HT22 cell viability. Accordingly, TBHQ at 0.05 and 0.1 nM, together with ML385 at 0.25 and 1 nM, was selected for subsequent experiments. To ascertain whether Slc2a1 expression is subject to Nrf2 regulation, protein levels of both molecules were examined by WB following pharmacological manipulation. Activation of Nrf2 by TBHQ elicited a concomitant elevation in Slc2a1 protein expression (Figure 5C,D). In contrast, pharmacological inhibition of Nrf2 signaling with ML385 resulted in a pronounced reduction in Slc2a1 abundance (Figure 5E,F). These observations suggest a positive regulatory association between Nrf2 activity and Slc2a1 expression in HT22 cells. This regulatory relationship was subsequently validated in vivo. WB analysis revealed that enhanced Nrf2 expression in hippocampal tissue was accompanied by a parallel increase in Slc2a1 protein levels (Figure 5G,H). Consistent findings were obtained from immunofluorescence staining of hippocampal sections (Figure 5I,J), while quantitative fluorescence analysis further substantiated this pattern (Figure 5K,L). Collectively, the results obtained from cell-based and animal experiments demonstrate that pharmacological modulation of Nrf2 activity is accompanied by corresponding changes in Slc2a1 expression, supporting a positive regulatory association between Nrf2 signaling and Slc2a1. However, these findings do not establish whether this relationship involves direct transcriptional regulation.

### 3.6. Slc2a1 Deficiency Is Associated with Mitochondrial Dysfunction in AD

**Figure 6:**
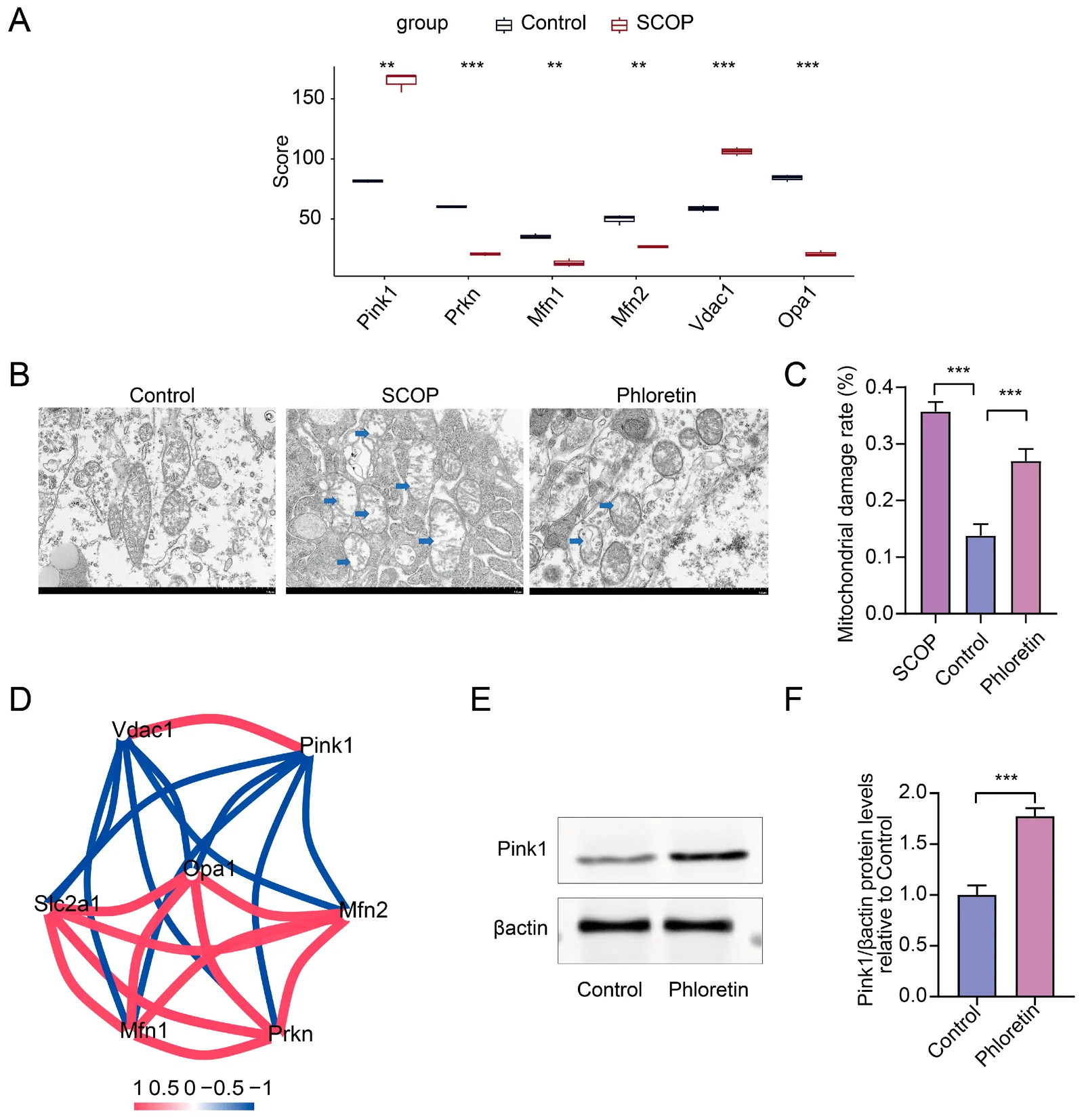
Slc2a1 suppression is associated with mitochondrial abnormalities. (**A**) Transcriptomic analysis of mitochondrial quality control-related genes in the Control and SCOP groups. (**B**) Representative transmission electron microscopy images illustrating mitochondrial ultrastructure in the Control, SCOP, and Phloretin-treated groups. Abnormal mitochondria are indicated by blue arrows. (scale bar = 10 μm). (**C**) Quantitative analysis of mitochondrial damage rates across experimental groups. (**D**) Correlation network depicting the association between Slc2a1 and mitochondrial quality control-related genes. Red lines indicate positive correlations, whereas blue lines indicate negative correlations. (**E**) Representative WB images showing Pink1 protein expression in the Control and Phloretin-treated groups. (**F**) Densitometric quantification of relative Pink1 protein expression levels. (** *p* < 0.01, *** *p* < 0.001).

To delineate the potential nexus between Slc2a1 dysregulation and downstream mitochondrial perturbations, transcriptomic profiling was integrated with in vivo experimental analyses. Initially, mitochondrial aberrations in SCOP-treated mice were interrogated across both molecular and ultrastructural dimensions. Transcriptomic evaluation revealed pronounced dysregulation of mitochondrial-associated transcripts: Pink1 and Vdac1 were significantly upregulated, whereas Prkn, Mfn1, Mfn2, and Opa1 were markedly downregulated (Figure 6A). To directly assess mitochondrial ultrastructure, hippocampal tissue was subjected to transmission electron microscopy, which unveiled characteristic pathological features in SCOP-treated mice, including mitochondrial swelling and cristae disruption, indicating a concurrent emergence of Slc2a1 downregulation and mitochondrial damage (Figure 6B,C). Correlation analysis between Slc2a1 and mitochondria-related transcripts revealed that Slc2a1 expression was positively correlated with Mfn1, Mfn2, Opa1, and Prkn, but negatively correlated with Pink1 and Vdac1. Notably, all these Spearman correlation coefficients exceeded 0.94 (Figure 6E). To further investigate the association between pharmacological inhibition of Slc2a1 and mitochondrial abnormalities, mice were treated with phloretin. Ultrastructural examination revealed that, unlike control with intact morphology and uniformly distributed organelles, Phloretin-treated group exhibited conspicuous mitochondrial defects, including compromised outer membrane integrity, disorganized cristae, and localized vacuolization (Figure 6C). Consistently, Western blot analyses demonstrated a marked elevation in Pink1 protein levels in Phloretin-treated cells (Figure 6F,G), indicative of heightened mitochondrial stress signaling. Collectively, these findings demonstrate that reduced Slc2a1 expression in SCOP-treated mice is accompanied by mitochondrial abnormalities and that pharmacological inhibition with phloretin is similarly associated with mitochondrial structural alterations and increased Pink1 expression. These findings support a potential functional relationship between Slc2a1 and mitochondrial homeostasis; however, they do not establish a Slc2a1-specific causal effect because potential off-target effects of phloretin cannot be excluded.

## 4. Discussion

Although the pathogenesis of AD is multifactorial and remains incompletely understood, increasing evidence indicates that oxidative stress represents one of the earliest pathological events driving neuronal dysfunction [14]. Persistent oxidative stress disrupts redox homeostasis, promotes mitochondrial injury, and ultimately contributes to progressive neuronal loss and cognitive impairment. Through integrative transcriptomic and bioinformatic analyses, we identified Slc2a1 as an Nrf2-associated candidate gene potentially related to mitochondrial dysfunction under oxidative stress conditions. Further investigation of the Nrf2/Slc2a1 axis may provide new insights into AD pathogenesis and identify potential therapeutic targets.

Oxidative stress serves as a central hub linking Aβ pathology, Tau pathology, neuroinflammation, and metal ion dyshomeostasis. Specifically, excessive ROS promotes the oxidative modification of amyloid precursor protein (APP), thereby facilitating Aβ deposition, while simultaneously activating kinases such as GSK3β and CDK5 to drive Tau hyperphosphorylation. Excessive ROS also activates microglia and astrocytes, triggering persistent neuroinflammation. These pathological alterations further exacerbate ROS generation, thereby establishing a self-perpetuating vicious cycle that ultimately culminates in neuronal injury [15,16]. SCOP model provides an experimental setting in which oxidative stress represents a predominant pathological insult, making it particularly suitable for investigating oxidative stress-associated molecular mechanisms [17,18]. In the present study, we first performed functional enrichment analysis of the differentially expressed genes identified by peripheral blood RNA sequencing between the Control and SCOP-treated mice. The results revealed that these differentially expressed genes were significantly enriched in oxidative stress-related pathways. Importantly, peripheral blood transcriptomics in the present study was used as a discovery strategy rather than as a direct surrogate for hippocampal gene expression. Peripheral blood may capture systemic molecular responses associated with the pathological state; however, its transcriptomic profile is influenced by circulating cell populations and systemic responses and therefore does not necessarily mirror molecular alterations in hippocampal neurons. Accordingly, the transcriptomic findings were primarily used to generate candidate genes, whose relevance to central pathology was subsequently evaluated through independent experiments in hippocampal tissue and HT22 cells. Consistent with these findings, SCOP-treated mice exhibited excessive ROS accumulation in the hippocampus, accompanied by reduced expression of the key antioxidant enzymes SOD2 and CAT, indicating that SCOP induced marked oxidative stress. These alterations were associated with neuronal damage and cognitive impairment [19].

As a master transcriptional regulator of the cellular antioxidant defense system, Nrf2 plays an indispensable role in preserving redox homeostasis in the brain [20]. Prolonged oxidative stress exerts a pathologically suppressive effect on both the expression and transcriptional activity of Nrf2 [21,22]. This may be associated with Nrf2 protein misfolding, aberrant ubiquitination, and subsequent degradation [23,24]. In the present study, sustained oxidative stress elicited by SCOP administration markedly suppressed hippocampal Nrf2 expression, whereas restoration of Nrf2 activity effectively attenuated neuronal injury and ameliorated cognitive deficits. Despite the well-established neuroprotective functions of Nrf2, its downstream regulatory landscape remains far from fully elucidated. Previous investigations have largely centered on canonical antioxidant targets, such as HO-1 and NQO1, while the molecular factors associated with Nrf2 signaling to the pathophysiological progression of AD remain incompletely characterized [25,26]. To further delineate the molecular mechanisms through which Nrf2 influences AD pathology, we integrated transcriptomic profiling with machine-learning algorithms and identified Slc2a1 as a candidate gene associated with Nrf2 signaling.

The Slc2a1 gene encodes glucose transporter 1 (GLUT1), a key transporter responsible for maintaining cerebral glucose uptake and energy metabolism [27]. Reduced GLUT1 expression has been consistently observed in the brains of patients with AD, leading to cerebral glucose hypometabolism and energy deficits, and may also contribute to tau hyperphosphorylation and Aβ deposition [28]. Previous studies have demonstrated that Aβ can suppress the Nrf2/GLUT1 signaling axis through SENP1-mediated stabilization of PUM2, resulting in impaired astrocytic glycolysis and aggravated neuronal apoptosis. Furthermore, they demonstrated that Nrf2 delays AD progression by transcriptionally upregulating GLUT1 expression in astrocytes [29]. Consistent with these observations, our findings demonstrate that pharmacological activation or inhibition of Nrf2 is accompanied by corresponding changes in Slc2a1 expression, supporting a positive regulatory association between Nrf2 signaling and Slc2a1 under AD-related pathological conditions. Nevertheless, the present data do not establish a direct transcriptional interaction between Nrf2 and Slc2a1.

Mitochondrial dysfunction has increasingly emerged as a central pathological nexus linking oxidative stress to neuronal loss in AD [30,31]. Oxidative stress not only compromises mitochondrial integrity and bioenergetic function but also promotes further mitochondrial ROS production, thereby establishing a self-perpetuating cycle that amplifies cellular injury [32]. The research team found that downregulation of Slc2a1 expression was accompanied by significant mitochondrial ultrastructural abnormalities and dysregulation of key factors regulating mitochondrial quality control. This is supported by existing reports: inhibiting GLUT1 expression can damage mitochondrial function, and the potential mechanism is that inhibiting GLUT1 can reduce mitochondrial glucose uptake and further disrupt mitochondrial membrane potential, leading to the accumulation of mitochondrial reactive oxygen species and causing damage to mitochondrial function [33]. Taken together, these findings support a potential association among oxidative stress, Nrf2 signaling, Slc2a1 expression, and mitochondrial abnormalities. On this basis, we propose that the Nrf2/Slc2a1 pathway may represent a potential mechanistic link between oxidative stress and mitochondrial dysfunction in AD-related pathological conditions. However, this proposed model requires further validation through genetic manipulation and rescue experiments.

Nevertheless, the SCOP model primarily recapitulates oxidative stress-associated cognitive impairment rather than the progressive neuropathological hallmarks of AD, such as amyloid-β deposition and tau hyperphosphorylation [34]. Therefore, validation of the Nrf2/Slc2a1 axis in transgenic AD models, including APP/PS1 and 5 × FAD mice, will be essential to establish its relevance across distinct pathological contexts. This represents an important direction for our future research. The Nrf2/Slc2a1 axis may play an important role in maintaining cerebral metabolic homeostasis in Alzheimer’s disease [35,36]. Unfortunately, metabolic parameters were not evaluated in the present study, elucidating whether the Nrf2/Slc2a1 axis contributes to neuronal metabolic adaptation under oxidative stress represents an important direction for future investigation. In addition, although pharmacological modulation of Nrf2 consistently altered Slc2a1 expression in both in vitro and in vivo experiments, the present study did not directly determine whether Nrf2 transcriptionally regulates Slc2a1. Further studies using approaches such as ChIP-qPCR and promoter-reporter assays are required to elucidate the precise regulatory mechanism between Nrf2 and Slc2a1. Finally, the mechanistic investigations in this study relied primarily on pharmacological interventions; however, the potential off-target effects of these compounds cannot be completely excluded. Future studies will employ genetic approaches, including Nrf2 or Slc2a1 knockdown/knockout and corresponding rescue experiments, to further establish target specificity and causal relationships.

In summary, the present study supports a potential functional relationship among oxidative stress, Nrf2 signaling, Slc2a1 expression, and mitochondrial dysfunction in the SCOP-induced AD model. Pharmacological modulation of Nrf2 was associated with corresponding changes in Slc2a1 expression, while pharmacological inhibition of Slc2a1 was accompanied by mitochondrial abnormalities. Further studies employing genetic manipulation and rescue experiments are required to establish the direct regulatory relationships and target-specific causal mechanisms underlying these observations.

## Data Availability

The datasets used and/or analysed during the current study are available from the corresponding author on reasonable request.

## Supplementary Materials

The following supporting information can be downloaded at: https://www.mdpi.com/article/10.3390/genes17091081/s1. Table S1. Informaion of differentially expressed genes.

## Abbreviations

The following abbreviations are used in this manuscript:

AD	Alzheimer’s disease
AUC	Area Under the Curve
CA1	Cornu Ammonis 1
CA3	Cornu Ammonis 3
CCK-8	Cell Counting Kit-8
DAPI	4′,6-diamidino-2-phenylindole
DEGs	Differentially Expressed Genes
DG	Dentate Gyrus
DHE	Dihydroethidium
GEO	Gene Expression Omnibus
GO	Gene Ontology
HE	Hematoxylin and Eosin
KEGG	Kyoto Encyclopedia of Genes and Genomes
MWM	Morris Water Maze
NOR	Novel Object Recognition
PVDF	Polyvinylidene Fluoride
ROC	Receiver Operating Characteristic
ROS	Reactive Oxygen Species
SCOP	Scopolamine
TBHQ	Tert-Butylhydroquinone
TEM	Transmission Electron Microscopy
WB	Western Blot
