# Supplementary material for: Identification and Validation of the Nrf2/Slc2a1 Axis in Alzheimer’s Disease
Source: Genes (Basel). 2026 Sep 8;17(9):1081. doi: 10.3390/genes17091081 (PMC13606258; doi:10.3390/genes17091081)
Supplement: Supplementary file 1 [file genes-17-01081-s001.zip › genes-4520604-supplementary.pdf]

Table S1. Information of differentially expressed genes

ID	logFC	P.Value	adj.P.Val
R3hdm4	-7.73	5.61E-08	0.000571529
Reep5	7.893666667	7.80E-08	0.000571529
Rps3l1	-1.10267	1.54E-07	0.000754303
Rps21	-8.100166667	3.03E-07	0.000969272
Ddx19b	2.216666667	3.31E-07	0.000969272
Ahrr	9.93	5.40E-07	0.001319626
Yif1a	-1.19	8.86E-07	0.001854995
Gid8	-6.244666667	4.50E-06	0.00824214
Fubp3	-1.074	5.53E-06	0.008646239
Vhl	1.008	5.90E-06	0.008646239
RT1N2	5.873333333	6.93E-06	0.009234843
Eif2b1	-1.019	7.72E-06	0.00943681
Klhl9	-1.253	1.05E-05	0.010386169
Wdfy1	-1.182666667	1.06E-05	0.010386169
St6gal1	-1.126333333	1.06E-05	0.010386169
Zfat	-4.726666667	1.62E-05	0.014826827
Slc35c1	-1.079666667	1.81E-05	0.015619044
Dok1	-1.059	2.13E-05	0.01734581
Hnrnpd	1.388933333	2.72E-05	0.020964316
Slc2a1	-13.00666667	2.87E-05	0.021052933
Prdm2	-1.148	3.02E-05	0.021096338
Ing2	1.141	3.34E-05	0.021632893
Cpsf1	-1.195	3.39E-05	0.021632893
Nucb1	6.833333333	3.56E-05	0.021759635
Kdm5c	-5.246666667	4.51E-05	0.026471534
Ppic	-2.505333333	6.13E-05	0.034584214
Snai2	-2.736666667	8.89E-05	0.048302358
Paox	-4.096666667	0.000115129	0.058162413
Wdr36	-9.863333333	0.000118527	0.058162413
Chrdl2	5.193333333	0.000119006	0.058162413
Cbfa2t3	-4.403333333	0.000133956	0.063257996
Tex261	4.393333333	0.000138061	0.063257996
Josd2	1.213333333	0.000145289	0.064552338
Klrb1b	1.186333333	0.000168885	0.072167707
Trib3	4.393333333	0.000172273	0.072167707
Shmt1	-1.011666667	0.000223993	0.090340778
Tspan33	3.023	0.000228722	0.090340778
Rarg	-1.042333333	0.000237337	0.090340778
Trib2	-5.716666667	0.000240301	0.090340778
Exoc3l2	-2.223333333	0.000256328	0.093956949
Gdf10	-3.356666667	0.000293161	0.1036702
Fmnl3	-1.012333333	0.000301541	0.1036702

Mt2A	16.87233333	0.000304039	0.1036702
Il4i1	-2.066666667	0.000326318	0.106336794
Cd2ap	-1.308666667	0.000328808	0.106336794
Serf2	8.808333333	0.000333617	0.106336794
Tsc22d4	-1.149	0.000356135	0.108835653
AC128848.1	14.31266667	0.000356303	0.108835653
Apoe	-3.6273	0.000380598	0.113884249
Piezo1	-5.683333333	0.000401096	0.117617464
Sox4	-3.623333333	0.00041621	0.119656234
Numa1	-9.086666667	0.000435404	0.119947383
Kctd11	-2.26	0.000438132	0.119947383
Hdac7	-5.71	0.000441765	0.119947383
Nnat	-9.473333333	0.000459783	0.119985088
Rpl3	9.041	0.000462145	0.119985088
Mcpt8l3	-3.006666667	0.000466454	0.119985088
Spout1	8.33	0.000522058	0.130568184
Cecr2	-1.324	0.000525407	0.130568184
Ccl24	-4.876666667	0.000543223	0.130907208
Commd7	2.284	0.000555725	0.130907208
Dlst	3.113333333	0.000561278	0.130907208
Ptms	4.763333333	0.000571004	0.130907208
Hdc	-1.101	0.000571413	0.130907208
Rdx	-9.816666667	0.000582875	0.131002807
C15h13orf42	5.263333333	0.0005897	0.131002807
Gimap4	-4.296666667	0.00060363	0.132095858
Mocs2	2.623333333	0.000618264	0.133200487
Tnrc18	-4.356666667	0.000627791	0.133200487
Ppfibp2	-3.446666667	0.000641368	0.133200487
B3gnt9	-2.743333333	0.000645017	0.133200487
Zfp36l1	-1.805333333	0.000654386	0.133258415
Amdhd2	9.533333333	0.000700245	0.139747525
Arih2	5.216666667	0.000705314	0.139747525
Col8a1	-1.823333333	0.000725023	0.141737197
Bcl7c	-5.246666667	0.000793276	0.153039604
Atn1	-3.566666667	0.000808058	0.153530838
Kank2	-7.093333333	0.000825066	0.153530838
Efs	-3.426666667	0.000827236	0.153530838
Fbxw4	-1.86	0.000852936	0.156321875
Runx2	-4.753333333	0.000878716	0.158539447
Marchf5	5.26	0.000896913	0.158539447
Rai1	-1.98	0.000906996	0.158539447
Timm21	8.403333333	0.000910695	0.158539447
Pkd1	-2.746666667	0.000921243	0.158539447
Vav2	-4.51	0.000929914	0.158539447

Nab2	-3.216666667	0.000950355	0.160162129
G3bp2	4.366666667	0.000968977	0.160406244
Rab20	7.063333333	0.000974734	0.160406244
Ppp1r9b	-1.646333333	0.000984624	0.160406244
Wnt4	2.253333333	0.00103984	0.167539953
Npr1	-2.38	0.001097602	0.174924425
Tbc1d10a	-4.013333333	0.001138394	0.179474496
Unc5b	-4.883333333	0.001153071	0.17985461
Rasgrp3	-3.883333333	0.001172295	0.180321229
St7l	-1.736666667	0.00118066	0.180321229
Ndnf	-2.336666667	0.001193007	0.180328616
Ier3	-1.594666667	0.001247231	0.182658409
Slc5a3	2.073333333	0.001247692	0.182658409
Eml2	-3.086666667	0.001264318	0.182658409
Vstm4	-2.003333333	0.001269079	0.182658409
Slc27a3	-4.843333333	0.001270711	0.182658409
Nagk	-1.83	0.001311547	0.186698043
Pitpnm2	-2.613333333	0.001326327	0.186986552
Fam219b	-3.46	0.00136336	0.18921108
Nr1h3	-3.67	0.001367915	0.18921108
Tamalin	-2.666666667	0.001405182	0.192549345
Ptprs	-5.126666667	0.001443309	0.195942514
Efna1	-3.543333333	0.00147695	0.198083003
Ptges3l1	-4.023333333	0.001492381	0.198083003
Smarcc2	-1.043	0.00151912	0.198083003
Srsf3	8.634	0.001531455	0.198083003
Gxylt2	-1.97	0.001533992	0.198083003
Etfa	1.029	0.001540135	0.198083003
Ggcx	-3.456666667	0.001601228	0.202507978
Ciao2b	-5.74	0.001602164	0.202507978
Llgl1	-2.426666667	0.001619913	0.203001443
Baiap2l1	-1.923333333	0.001640745	0.203869534
Aff3	-1.593333333	0.001677669	0.205862391
Traf5	-2.36	0.001705239	0.205862391
Abi3	-4.063333333	0.001718542	0.205862391
Zbtb45	-1.336666667	0.00172167	0.205862391
Marcks	-8.436666667	0.001732266	0.205862391
Btla	-3.866666667	0.001752769	0.205862391
Gimap8	-1.376666667	0.001755067	0.205862391
Klhl18	-1.973333333	0.001769688	0.205929839
Ccn4	-4.9	0.001813136	0.207882134
Kdr	-4.633333333	0.0018572	0.207882134
Satb2	-4.693333333	0.001873529	0.207882134
C5ar2	-7.186666667	0.001896571	0.207882134

Myo1e	-3.513333333	0.001899618	0.207882134
Arsl	-2.676666667	0.001908412	0.207882134
Rgs2	2.065	0.00192774	0.207882134
Cog7	-2.506666667	0.001946005	0.207882134
Stmp1	7.526666667	0.001950345	0.207882134
Smad1	-3.23	0.00196964	0.207882134
Snx33	-2.75	0.001973954	0.207882134
Per1	2.127666667	0.001991932	0.207882134
Prr12	-1.62	0.002019861	0.207882134
Ano10	2.936666667	0.002020399	0.207882134
Rapgef3	-1.966666667	0.002070142	0.207882134
Pde6d	-3.2	0.002106202	0.207882134
Sap30	7.216666667	0.002112585	0.207882134
Chst15	-2.646666667	0.002145572	0.207882134
Gfm1	3.56	0.00217215	0.207882134
Tjp2	-5.18	0.002185131	0.207882134
AABR07051787.1	2.43	0.002196439	0.207882134
Fcmr	-3.58	0.002201258	0.207882134
Ccdc167	-1.265666667	0.002215361	0.207882134
Kdm5b	-2.273333333	0.002215363	0.207882134
Myo1c	-1.064	0.002225052	0.207882134
Clec11a	-2.509	0.002225319	0.207882134
Zc3h4	-5.43	0.002227927	0.207882134
Golga3	-1.533333333	0.002242756	0.207882134
Atp5pb	5.632333333	0.002262816	0.207882134
Rgs3	-2.167666667	0.002264181	0.207882134
Shox2	-4.37	0.00227426	0.207882134
Irf7	-9.658	0.002285882	0.207882134
Emc3	8.606666667	0.002287993	0.207882134
Hdgfl2	-4.836666667	0.002310808	0.207882134
Col6a2	-3.772	0.002317196	0.207882134
Dctn3l1	4.26	0.002319073	0.207882134
Slfn5	-1.572333333	0.002325584	0.207882134
Klf1	9.579333333	0.002338175	0.207882134
Hnrnpa0	-1.19	0.002339418	0.207882134
Ddb1	-1.893	0.002370106	0.20808066
Klhl22	8.386666667	0.002374888	0.20808066
Cntf	2.296666667	0.002384228	0.20808066
Akr1b8	-2.37	0.002402296	0.208416976
Klhl24	-4.176666667	0.002430544	0.209627266
Rab42	-2.396666667	0.002481067	0.210407641
Fam171a2	-1.753333333	0.002483685	0.210407641
Rbm14	-5.866666667	0.00249583	0.210407641
Lzts2	-5.176666667	0.002496994	0.210407641

Ifngr1	3.511333333	0.002525756	0.211615079
Slamf9	-7.876666667	0.002605841	0.214144046
Serpinh1	-7.389666667	0.002612066	0.214144046
Irx5	-2.393333333	0.002638255	0.214144046
Sp7	-4.56	0.002638343	0.214144046
Chd4	-1.665666667	0.002643983	0.214144046
Alpl	-3.511333333	0.002652277	0.214144046
Dusp7	-3.233333333	0.002658179	0.214144046
Sh3d19	-2.413333333	0.002712375	0.215612856
Vdac2	1.798666667	0.002714512	0.215612856
Osbpl7	-2.12	0.002740813	0.215612856
Procr	-6.006666667	0.002749411	0.215612856
Dok4	-1.66	0.002761289	0.215612856
Ptprd	-5.253333333	0.002764644	0.215612856
Engase	-2.26	0.002804922	0.216790681
Ahnak2	-2.446666667	0.002823294	0.216790681
Ccr5	-3.21	0.002828343	0.216790681
Tmem104	-2.933333333	0.002854379	0.216790681
Paqr6	-2.57	0.002858515	0.216790681
Pcmt1	1.791333333	0.002871365	0.216790681
Adamts2	-4.976666667	0.002883248	0.216790681
Slc45a3	-3.093333333	0.00292582	0.218101343
Rxra	-1.671333333	0.00293043	0.218101343
Dpysl2	-8.706666667	0.002949088	0.218381438
Inpp1	-1.48	0.002982017	0.21888485
Rfxank	-1.396666667	0.002985743	0.21888485
Rgs1	2.558	0.003072233	0.219460908
Prkag1	-3.07	0.003077146	0.219460908
Hs1bp3	-1.866666667	0.003077428	0.219460908
Zbtb7b	-8.34	0.003088521	0.219460908
Angpt2	-4.486666667	0.003107496	0.219460908
Fgfr3	-5.266666667	0.003108305	0.219460908
Cabin1	-1.886666667	0.003124586	0.219460908
Zfp608	-2.25	0.003128057	0.219460908
Tns3	-1.069	0.003135505	0.219460908
Crem	1.242333333	0.003143281	0.219460908
mrpl24	-2.846666667	0.003188611	0.220708878
Sap30l	-3.363333333	0.003191262	0.220708878
Eif2s2	4.229	0.003222232	0.220925218
Tmem98	-2.196666667	0.003240723	0.220925218
Sppl2b	-3.176666667	0.003292201	0.220925218
Spats2l	-2.183333333	0.003297099	0.220925218
Rnf7	8.11	0.003302404	0.220925218
Pcp4l1	-6.043333333	0.003318917	0.220925218

Septin11	-8.83	0.003320186	0.220925218
Itpr3	-3.44	0.003347573	0.220925218
Itgb5	-1.819333333	0.003360073	0.220925218
Ano8	-3.84	0.003377977	0.220925218
Dlg3	-1.856666667	0.003379866	0.220925218
Gpx7	-6.433333333	0.003393289	0.220925218
Alg2	-1.43	0.003394819	0.220925218
Mrpl4	1.455666667	0.00340534	0.220925218
Pacsin1	-2.076666667	0.00342296	0.221090048
Mrfap1	-7.043333333	0.00344011	0.221223246
Zfpm1	-1.558	0.003455495	0.22124222
Slco3a1	-3.476666667	0.003473655	0.221437946
Vps18	-1.49	0.003506597	0.221526032
Prickle3	-2.483333333	0.003507923	0.221526032
Hapstr1	3.806666667	0.003536872	0.221526032
Lyl1	-1.570666667	0.00354719	0.221526032
Pgm3	-1.93	0.003550581	0.221526032
Fam78a	-2.636666667	0.003590466	0.223065324
Kdm2a	-6.863333333	0.003631695	0.223660307
Mmp25	4.523333333	0.003638725	0.223660307
Btbd6	-4.593333333	0.003647519	0.223660307
Nmnat3	9.216666667	0.003680215	0.223660307
Sh2b2	4.07	0.003687408	0.223660307
Tcim	3.04	0.003705118	0.223660307
Tcof1	-3.326666667	0.003746962	0.223660307
Oas1b	-1.036666667	0.003767449	0.223660307
Pip4p2	-1.633333333	0.003776834	0.223660307
C2h3orf80	-1.416666667	0.003780629	0.223660307
Nherf1	-6.93	0.003782573	0.223660307
Atp8b2	-3.913333333	0.003783096	0.223660307
Bcar3	-4.57	0.003878687	0.227794011
Rig1	-3.61	0.003884842	0.227794011
Fbxl8	-1.396666667	0.003903844	0.227794011
Dlx5	-3.893333333	0.003915161	0.227794011
Ticam1	-1.766666667	0.003937042	0.228161712
Bahd1	-4.133333333	0.003978282	0.228859667
Adamts5	-2.816666667	0.003980304	0.228859667
Plekha5	-3.663333333	0.004027766	0.230469037
Golga5	4.636666667	0.004039731	0.230469037
Hnrnpa3	2.82	0.004093113	0.232172715
Ppp1r2	6.033333333	0.004113887	0.232172715
Tmem51	-2.06	0.004126551	0.232172715
Galnt3	-1.853333333	0.004132934	0.232172715
Pura	-1.753333333	0.004162807	0.232593129

Cenpv	-2.25	0.004172145	0.232593129
Src	-4.186666667	0.004219581	0.234256173
Ephb4	-3.606666667	0.00423393	0.234256173
Fgfr2	-6.336666667	0.00426159	0.234900098
Ldlrad4	-2.243333333	0.00428634	0.235265662
Frrs1l	-1.716666667	0.004300314	0.235265662
Rn18s	18.80366667	0.004351585	0.237185673
Ccdc102a	2.193333333	0.004381274	0.237227105
RT1T244	-2.646333333	0.004397481	0.237227105
Sec31a	-8.393333333	0.004407625	0.237227105
Mob4	5.206666667	0.004430935	0.237227105
Ric1	-1.846666667	0.004442348	0.237227105
Pik3c2b	-1.683333333	0.0044708	0.237227105
Tnks	-1.786666667	0.004513245	0.237227105
Abcg2	4.4	0.004518124	0.237227105
Ear1	-7.481666667	0.004565288	0.237227105
Camk1	-6.12	0.004587941	0.237227105
Pogz	-1.62	0.004600057	0.237227105
Dvl2	-2.146666667	0.004639449	0.237227105
Gapvd1	-3.553333333	0.004656008	0.237227105
Jade2	-1.853333333	0.00465805	0.237227105
Cttn	-6.956666667	0.004709036	0.237227105
Sbf1	-4.013333333	0.004729806	0.237227105
Cic	-5.026666667	0.0047375	0.237227105
Acer2	3.66	0.004748604	0.237227105
Kmt2a	-2.613333333	0.004753071	0.237227105
Ptger4	3.06	0.004753348	0.237227105
LOC134478918	-1.5	0.004760379	0.237227105
Nup210l	-2.32	0.004760844	0.237227105
Morc2	-1.863333333	0.00476866	0.237227105
Tjp1	-4.753333333	0.004783534	0.237227105
Fam114a1	-8.696666667	0.00481426	0.237227105
Rasl11a	-1.186666667	0.004840106	0.237227105
Mtcl2	-1.693333333	0.004848839	0.237227105
Anapc16	9.473333333	0.004880571	0.237227105
Smim3	7.43	0.004902114	0.237227105
Ctdspl2	7.416666667	0.004905031	0.237227105
Fgf12	1.29	0.00491351	0.237227105
Sh3bp2	-6.333333333	0.004935879	0.237227105
R3hdm2	-1.663666667	0.004970632	0.237227105
C1qa	-7.199333333	0.004995391	0.237227105
Tbl2	-2.993333333	0.005007255	0.237227105
Zmynd8	-8.843333333	0.005010243	0.237227105
Ophn1	-2.006666667	0.005024943	0.237227105

Fam120b	-2.48	0.005033415	0.237227105
Podxl	-4.146666667	0.005072899	0.237227105
Irx3	-2.706666667	0.005093381	0.237227105
Pcgf2	-1.99	0.005096499	0.237227105
Cfap418	-1.75	0.005097034	0.237227105
Pgp	2.85	0.005106817	0.237227105
Psmc6	1.223333333	0.005121674	0.237227105
Tbc1d10b	-4.79	0.005125951	0.237227105
Wnk1	-6.553333333	0.00513981	0.237227105
Swap70	-6.353333333	0.005165078	0.237227105
Smad6	-2.29	0.005165261	0.237227105
Pax5	-1.085333333	0.005166945	0.237227105
Dennd4b	-8.35	0.005194498	0.237227105
RGD1559575	-7.523333333	0.005203687	0.237227105
Cul2	1.923333333	0.005221753	0.237227105
Mdn1	-1.183333333	0.005224964	0.237227105
Bcl9l	-2.51	0.005238507	0.237227105
Strn4	-3.04	0.00532767	0.237227105
Fam3c	-4.584	0.00533999	0.237227105
Zfp217	-8.316666667	0.005354033	0.237227105
C1qc	-9.618	0.005376576	0.237227105
Taok3	-6.153333333	0.005382793	0.237227105
Bfar	2.493333333	0.005392928	0.237227105
Zfp142	-2.506666667	0.005400423	0.237227105
Fam169b	-1.09	0.00541026	0.237227105
Ubr4	-4.29	0.005414559	0.237227105
Akt1s1	-4.41	0.005422311	0.237227105
Emc2	1.23	0.005448231	0.237227105
Tmem214	-3.82	0.00546222	0.237227105
Fgfr1	-2.285333333	0.005476492	0.237227105
Six5	-1.773333333	0.005478027	0.237227105
Prrc2b	-5.07	0.005482235	0.237227105
Nmnat1	-1.963333333	0.005484926	0.237227105
Hsph1	-7.906666667	0.005535561	0.238081208
Tarbp1	-1.76	0.00553715	0.238081208
Zfyve26	-1.246666667	0.005599657	0.238570813
Cfd	-1.4568	0.005642266	0.238570813
Vasn	-4.54	0.005661936	0.238570813
Neurl1b	-1.84	0.005665735	0.238570813
Ccng2	4.83	0.005667891	0.238570813
Hr	-2.846666667	0.005678962	0.238570813
Cad	-2.833333333	0.005710983	0.238570813
Mmp14	-2.583333333	0.005714763	0.238570813
P3h3	-7.046666667	0.005722759	0.238570813

Cx3cr1	-1.82	0.005740709	0.238570813
Ceacam1	2.977333333	0.005748168	0.238570813
Cst3	-1.325743333	0.005773293	0.238570813
Pdgfrb	-8.83	0.005782096	0.238570813
Cdr2l	-1.513333333	0.005790405	0.238570813
Tspan18	-2.076666667	0.005792607	0.238570813
Selenom	-2.265666667	0.005810395	0.238633073
Klf7	-1.566666667	0.005833283	0.238903916
Cebpa	-6.31	0.005867623	0.23964093
Rftn1	-3.16	0.005922126	0.240916751
Wdr45	3.173333333	0.005931725	0.240916751
Psmd6	1.434333333	0.005953863	0.24114789
Pprc1	-2.056666667	0.006024446	0.241886835
Gata2	-4.336666667	0.006028055	0.241886835
Alkbh3	5.086666667	0.006038251	0.241886835
Btf3l4	2.676666667	0.006066717	0.241886835
Flnb	-4.443333333	0.006080695	0.241886835
Prrt1	-2.003333333	0.006084779	0.241886835
Nceh1	-4.533333333	0.006109814	0.241886835
Coq10b	3.588	0.006114261	0.241886835
Mgat4b	-8.243333333	0.006143836	0.241886835
Castor1	-1.32	0.006146537	0.241886835
Crtap	-9.576666667	0.00615358	0.241886835
Slco4a1	-2.226666667	0.006199837	0.242991395
Iqce	-1.043333333	0.006214826	0.242991395
Slamf7	-1.656666667	0.006248989	0.243677343
Deaf1	-3.493333333	0.006270122	0.243767826
Ifi44	-7.313333333	0.006301773	0.243767826
Drc1	4.643333333	0.006305257	0.243767826
Trappc12	-2.543333333	0.00632584	0.243767826
Erf	-2.866666667	0.006334439	0.243767826
Ptpn23	-1.78	0.00636093	0.244146492
Acaca	-2.526666667	0.006385388	0.244445329
Tiparp	7.213333333	0.006407513	0.244653544
Adprh	-2.32	0.006446382	0.244984925
Dync1h1	-5.39	0.006456863	0.244984925
Cdh11	-7.376666667	0.006511462	0.244984925
Selenot	4.036666667	0.006512208	0.244984925
Cntnap1	-1.95	0.006525355	0.244984925
Tax1bp3	-6.026666667	0.006540463	0.244984925
Cbr3	-2.09	0.006550321	0.244984925
Setd1b	-2.936666667	0.006566538	0.244984925
Prrx2	-4.44	0.00658954	0.244984925
Jun	-1.199	0.006599786	0.244984925

Dnah10	-1.466666667	0.006662406	0.244984925
Slc22a18	-1.44	0.006672595	0.244984925
Akr7a2	-7.073333333	0.006682797	0.244984925
Lrp4	-5.93	0.00669225	0.244984925
Pgghg	-3.19	0.006704079	0.244984925
Mvb12a	-8.136666667	0.006708183	0.244984925
Smo	-1.533333333	0.006715342	0.244984925
Mfsd3	-2.04	0.006716951	0.244984925
Zfp467	-1.76	0.006764368	0.246102148
Ifi27l2a	-9.224666667	0.006793366	0.246333491
Wiz	-1.83	0.006804328	0.246333491
Ebf1	-7.276666667	0.006829079	0.2465299
Pkn3	-1.313333333	0.006843382	0.2465299
Ankrd50	-1.173333333	0.006864524	0.246685413
Rufy3	-8.82	0.006915823	0.247581071
Dlg4	-1.156666667	0.006957878	0.247581071
Lipa	-1.473333333	0.006972698	0.247581071
Palm	-1.083333333	0.006978025	0.247581071
Prickle1	-1.313333333	0.006998013	0.247581071
Ddit4	1.226333333	0.007029379	0.247581071
Heg1	-2.17	0.007082172	0.247581071
Ubald2	1.067333333	0.007092911	0.247581071
Shroom4	-1.54	0.007100576	0.247581071
Ctbp2	-5.596666667	0.007105407	0.247581071
Zfp507	-1.736666667	0.007106213	0.247581071
Ctdsp2	-1.019	0.007126278	0.247581071
B3galnt1	-3.776666667	0.007131418	0.247581071
Rasgrp1	-1.95	0.007132467	0.247581071
Tmx4	1.210333333	0.007154099	0.247581071
Rab23	-2.87	0.007159704	0.247581071
Macir	1.338	0.007189692	0.247581071
Guf1	1.72	0.007193394	0.247581071
Plekhg3	-2.546666667	0.007227817	0.248183246
Abcb1b	5.423333333	0.007288005	0.24966526
Sybu	-1.146666667	0.007328629	0.250228678
LOC120100687	-1.096666667	0.007362301	0.250228678
Serpinb2	3.97	0.007384653	0.250228678
Ppm1m	-2.133333333	0.007399565	0.250228678
Pdgfrl	-1.869666667	0.007406826	0.250228678
Bdp1	-1.483333333	0.007484104	0.250228678
Slc22a23	-2.88	0.007495766	0.250228678
Hyou1	-7.253333333	0.007512735	0.250228678
Ppp2r2d	6.256666667	0.007533127	0.250228678
Fibp	-4.453333333	0.007555969	0.250228678

Aldh4a1	-2.13	0.007573999	0.250228678
C15h14orf93	-1.666666667	0.007582209	0.250228678
Dalrd3	-2.136666667	0.007609973	0.250228678
Gimap9	-1.95	0.007624376	0.250228678
Lxn	-6.553333333	0.007648144	0.250228678
Cep131	-2.95	0.007661704	0.250228678
Fzd2	-1.243333333	0.007663738	0.250228678
Tor4a	-3.24	0.00767496	0.250228678
Dgki	-3.56	0.007695587	0.250228678
Npas2	-1.546666667	0.007699372	0.250228678
Ndufs4	1.150666667	0.007717062	0.250228678
Fat1	-3.736666667	0.007717414	0.250228678
Brd3	-2.443333333	0.007718331	0.250228678
S100pbp	-3.303333333	0.007735282	0.250228678
Gnai1	-4.65	0.00774818	0.250228678
Ifrd1	1.543	0.007784387	0.250845471
Ptk7	-2.623333333	0.007821511	0.251174188
Myct1	-4.193333333	0.007832596	0.251174188
Fst	1.983333333	0.007848824	0.251174188
Sec14l1	-6.513333333	0.007872108	0.251174188
Selenon	-6.293333333	0.007880243	0.251174188
Dync2i2	-1.34	0.007970895	0.253512494
Abca2	-2.123333333	0.008105516	0.256271351
Nrp2	-1.218333333	0.008119537	0.256271351
Luzp1	-1.373333333	0.008143438	0.256271351
Dhrs9	2.17	0.008164672	0.256271351
Ndufb9	4.796666667	0.008180914	0.256271351
Adissp	-7.576666667	0.008190798	0.256271351
Bcat1	1.844666667	0.008225696	0.256271351
Ypel5	4.857333333	0.008255618	0.256271351
Aup1	8.28	0.008259805	0.256271351
Tram2	-2.926666667	0.008262318	0.256271351
Pard6g	-4.406666667	0.008281856	0.256271351
Tmem94	-2.516666667	0.008292009	0.256271351
Dnal1	-1.293333333	0.008310352	0.256271351
Tshz1	-1.966666667	0.008321864	0.256271351
Zfp532	-1.546666667	0.008322166	0.256271351
Farp2	-2.046666667	0.008337296	0.256271351
Tecpr1	-1.66	0.008387831	0.257032586
Cep170	-4.55	0.008397122	0.257032586
Nop2	-4.84	0.008422099	0.257089123
Kcnk5	-1.023333333	0.008434038	0.257089123
Nup214	-2.016666667	0.008487544	0.257435844
Adgra3	-2.76	0.008505533	0.257435844

Pxdn	-5.31	0.008511461	0.257435844
LOC103692127	-2.13	0.008525325	0.257435844
Mzb1	-2.653333333	0.008533203	0.257435844
Aldh18a1	-3.36	0.008604213	0.258176103
Ssh3	-1.823333333	0.008641756	0.258176103
Mapkbp1	-1.106666667	0.008644065	0.258176103
2010315B03Rikl	-1.603333333	0.008661981	0.258176103
B4galt4	-1.31	0.008737698	0.258176103
Mtss2	-3.23	0.008746507	0.258176103
Dnal4	-1.473333333	0.008782213	0.258176103
Nolc1	-5.343333333	0.008848741	0.258176103
Rspo1	-1.7	0.008848944	0.258176103
Fam50a	-7.183333333	0.008855622	0.258176103
Dennd5b	-1.373333333	0.00886671	0.258176103
Dync2li1	-2.056666667	0.008876062	0.258176103
Pcgf3	-1.333333333	0.008881369	0.258176103
Znfx1	-3.283333333	0.008895752	0.258176103
Arhgef25	-2.976666667	0.008899179	0.258176103
Mrps16	2.68	0.008923092	0.258176103
C1h19orf33	-1.031333333	0.008929253	0.258176103
Tstd2	-1.91	0.008938609	0.258176103
Cebpb	2.49	0.008939981	0.258176103
Cryz	-1.276666667	0.008946844	0.258176103
Sehl1	-1.753333333	0.008947595	0.258176103
Raph1	-1.626666667	0.008988452	0.258176103
Cd79b	-2.705	0.008994476	0.258176103
Calhm2	-3.523333333	0.009014989	0.258176103
Kitlg	-9.163333333	0.009028721	0.258176103
Serpinb8	-1.776666667	0.009032017	0.258176103
Iqcd	-3.816666667	0.00903317	0.258176103
Actn4	-2.304	0.009056384	0.258336004
Rabgap1	-2.103333333	0.009140293	0.259460644
Ywhag	-1.537	0.009161191	0.259460644
Septin10	-2.003333333	0.009178509	0.259460644
Ldlr	-6.633333333	0.009179958	0.259460644
Cmah	-2.803333333	0.009184291	0.259460644
Fut7	-1.496666667	0.009207167	0.259606698
Tnfrsf13c	-1.746666667	0.009263613	0.26069693
Klhl20	-1.476666667	0.009284421	0.260762673
Plekhg2	-2.79	0.009301519	0.260762673
Sox18	-5.013333333	0.009381762	0.261076067
Creb3l1	-3.83	0.009382614	0.261076067
Ttc1	7.816666667	0.009396921	0.261076067
Ahdc1	-1.853333333	0.009406161	0.261076067

Zfp653	-1.453333333	0.009425184	0.261076067
Siah1	3.606666667	0.009434373	0.261076067
Cul7	-1.7	0.009437878	0.261076067
Cbx8	-1.81	0.009502637	0.261076067
Myadm	-1.275	0.009506928	0.261076067
Bach2	-3.373333333	0.00952221	0.261076067
Mafb	-5.56	0.009525881	0.261076067
Zcchc14	-2.446666667	0.009526374	0.261076067
Tnrc6c	-2.203333333	0.009605887	0.262563812
Ankrd42	-1.216666667	0.009644562	0.262563812
G0s2	-8.776666667	0.009657918	0.262563812
Gpx8	-8.98	0.00970108	0.262563812
Apobec1	-7.016666667	0.009741438	0.262563812
Sdc4	1.502666667	0.009763469	0.262563812
Utp25	-1.603333333	0.009805756	0.262563812
Col12a1	-8.523333333	0.009840811	0.262563812
Uap1l1	-3.116666667	0.009862787	0.262563812
Il1a	-1.096666667	0.009877396	0.262563812
Gucy1a1	-4.68	0.009899834	0.262563812
Zfp428	-1.383333333	0.009929531	0.262563812
Ndufb6	-4.013333333	0.009932562	0.262563812
Dio2	1.406666667	0.009933451	0.262563812
Klf2	-1.232333333	0.009934058	0.262563812
Taf4	-3.436666667	0.009940703	0.262563812
Bicra	-2.866666667	0.00994384	0.262563812
Lax1	1.933333333	0.009945168	0.262563812
Zbtb4	-2.846666667	0.009949068	0.262563812
Cd9	-4.772333333	0.009964589	0.262563812
Ifit1bl	-2.722	0.009980227	0.262563812
Gask1a	-1.033333333	0.009984839	0.262563812
LOC134482331	-1.143666667	0.009992539	0.262563812
Coro2b	-1.36	0.010016059	0.262711021
Thbs2	-8.423333333	0.010050949	0.263155379
Cep170b	-1.826666667	0.010083076	0.263525955
Rab11fip1	-2.2	0.010109507	0.263542725
Frrs1	-3.413333333	0.010137203	0.263542725
Rdh10	-1.46	0.010145239	0.263542725
Slc15a2	-1.36	0.010158919	0.263542725
Mettl16	-1.936666667	0.01017359	0.263542725
Cndp1	-1.556666667	0.010204302	0.263872104
Zfp335	-1.703333333	0.010260792	0.264822859
Trappc14	-3.463333333	0.010294723	0.264822859
Dock1	-2.393333333	0.01033284	0.264822859
Tdrp	-1.733333333	0.010346086	0.264822859

Lmf2	-5.38	0.010381192	0.264822859
C1qtnf5	-6.826666667	0.01040499	0.264822859
Macf1	-1.178333333	0.010431352	0.264822859
Srebf2	-2.59	0.010437193	0.264822859
Bcorl1	-1.656666667	0.010445456	0.264822859
Rtp3	-1.176666667	0.010495113	0.264822859
Chd9	-1.83	0.010504709	0.264822859
Olfml3	-1.332333333	0.010519871	0.264822859
Bcl2l12	-3.7	0.010523507	0.264822859
Acly	-1.309666667	0.010524805	0.264822859
Bcor	-3.813333333	0.010538451	0.264822859
Ccrl1l	-2.036666667	0.010556916	0.264822859
Hbs1l	8.793333333	0.010595117	0.264822859
Herpud1	7.023333333	0.010608221	0.264822859
Khdrbs3	-2.33	0.010654093	0.264822859
Atf7ip	-7.436666667	0.010668828	0.264822859
Chpf	-3.833333333	0.010673343	0.264822859
Fbxw5	-2.7	0.010675091	0.264822859
Mtrf1l	-1.566666667	0.010687416	0.264822859
Lfng	-7.736666667	0.010714842	0.264822859
Irf4	-3.256666667	0.010718067	0.264822859
Gigyf2	-2.77	0.010718697	0.264822859
Tmem184b	-3.19	0.010770432	0.264822859
Lpin3	-1.45	0.010806517	0.264822859
Prrc1	-3.123333333	0.010808941	0.264822859
Fam167a	-1.426666667	0.010818413	0.264822859
Tmem176a	-1.073666667	0.010831894	0.264822859
Itga5	-4.19	0.010869318	0.264822859
Igfbp4	-1.482333333	0.010888086	0.264822859
Il18rap	1.084666667	0.01089202	0.264822859
Plod3	-3.753333333	0.010933836	0.264822859
Stard9	-1.07	0.010941409	0.264822859
Pdcd5	1.229666667	0.010972024	0.264822859
Camsap3	-1.286666667	0.010980036	0.264822859
Sv2a	-1.236666667	0.010988518	0.264822859
Adcy6	-4.123333333	0.011000289	0.264822859
Chd3	-1.315666667	0.011015659	0.264822859
Slc25a1	-2.986666667	0.011017729	0.264822859
Arhgap21	-1.866666667	0.011054695	0.264879806
Ramp3	9.246666667	0.01107505	0.264879806
Esd	4.65	0.011117496	0.264879806
Bcs1l	-1.746666667	0.011153491	0.264879806
Eif5	3.212666667	0.011156148	0.264879806
Arhgef10	-1.96	0.011162537	0.264879806

Acsl4	1.104666667	0.01116415	0.264879806
Mbd6	-5.056666667	0.011164624	0.264879806
Mrpl45	1.317333333	0.011195082	0.265123674
Slc38a10	-4.86	0.011211683	0.265123674
Flt3lg	-1.726666667	0.011240718	0.265123674
Ptrh1	-3.413333333	0.011247233	0.265123674
Ccnq	-1.673333333	0.011315066	0.26585604
Pip5k1c	-3.206666667	0.011325523	0.26585604
H1f0	4.695666667	0.011332698	0.26585604
Sema6a	-1.426666667	0.011383349	0.266101579
Zfp503	-1.323333333	0.011400408	0.266101579
Zc3hc1	3.72	0.011416614	0.266101579
Rubcn	-3.043333333	0.011430717	0.266101579
Tgfb1i1	-5.253333333	0.011449616	0.266101579
Apba3	-4.326666667	0.01145206	0.266101579
Dkk2	-1.123333333	0.011511574	0.266639343
Wdr41	9.77	0.011564573	0.267347652
Pds5b	-3.11	0.011578622	0.267347652
Itga6	-1.022333333	0.011624819	0.267889228
Lrrc15	-1.39	0.011656197	0.267889228
Rpl7a	1.946666667	0.01165689	0.267889228
Hcfc1	-5.876666667	0.011685546	0.268127501
Rcn1	-1.363333333	0.01173373	0.268812427
Tbc1d12	-1.4	0.011772967	0.269290547
Micall1	-1.65	0.011814889	0.269828514
LOC120101770	-6.436666667	0.01184149	0.270015445
Gse1	-7.36	0.011882247	0.270524078
Polr1b	-2.376666667	0.011946043	0.271329525
P4ha2	-4.453333333	0.011954638	0.271329525
Aldh1l2	-1.266666667	0.011994396	0.271329525
Nherf2	-7.673333333	0.011997257	0.271329525
Srf	-3.406666667	0.012010153	0.271329525
Sertad4	-1.81	0.012040827	0.271604014
Ipo9	-5.513333333	0.012072016	0.271696516
Ski	-5.803333333	0.012099945	0.271696516
Xylt1	-1.18	0.01210052	0.271696516
Pld2	-2.353333333	0.01213299	0.271999862
Lima1	-5.346666667	0.012168821	0.271999862
Prrx1	-1.322333333	0.012169684	0.271999862
Klf11	4.41	0.012198091	0.272219805
Mpzl1	-8.253333333	0.012218613	0.272263389
Bicral	-2.683333333	0.012248737	0.272332598
Asap3	-1.433333333	0.012277839	0.272332598
Kidins220	-4.496666667	0.012283359	0.272332598

Cspg4	-1.863333333	0.012301545	0.272332598
Il1r1	3.28	0.012334943	0.272332598
Plcb3	-2.97	0.012338235	0.272332598
Loxl1	-3.586666667	0.012364574	0.272332598
Smad7	-3.083333333	0.012379859	0.272332598
Arfip2	-6.86	0.012416275	0.272332598
Fam167b	-4.766666667	0.01243616	0.272332598
Tubb1	-1.484666667	0.012449183	0.272332598
Osbpl5	-3.333333333	0.012456923	0.272332598
Xpr1	-2.253333333	0.012465184	0.272332598
Adam12	-2.916666667	0.012481756	0.272332598
Anapc2	-2.383333333	0.012518279	0.272394245
Capn15	-1.93	0.012521738	0.272394245
Kctd10	-6.2	0.012668589	0.274773449
Notch2	-4.573333333	0.012731112	0.275702226
Itpkb	-5.14	0.012761098	0.275702226
Ralgds	-4.9	0.012794167	0.275702226
Arid5a	3.963333333	0.012803277	0.275702226
Kif13b	-1.336666667	0.012845065	0.275702226
Tfcp2	-1.21	0.012847405	0.275702226
Pparg	-8.123333333	0.012861319	0.275702226
Slc2a10	-1.12	0.012861842	0.275702226
Fbxw9	-1.216666667	0.01288132	0.275716666
Cd36ps1	3.781666667	0.012964937	0.277081443
Kazald1	-3.396666667	0.013001233	0.277081443
Rps6ka3	-1.903333333	0.013001776	0.277081443
Bad	-2.63	0.013038778	0.277466701
Zbtb48	-1.376666667	0.013062106	0.277560278
Tep1	-2.716666667	0.013094048	0.277836369
Arhgef15	-1.343333333	0.013130481	0.277911301
Mk1	-1.666666667	0.013135488	0.277911301
Eeig1	-2.64	0.013194812	0.277929467
Proser3	-1.266666667	0.013199658	0.277929467
Camsap1	-1.556666667	0.013206955	0.277929467
Mme	-4.11	0.013248479	0.277929467
Acin1	-1.078	0.013248647	0.277929467
Nuggc	-1.01	0.013288191	0.27823869
Pfkfb3	2.201	0.013302777	0.27823869
Pcyox1l	-1.496666667	0.013401506	0.279904381
Dnaaf9	-1.413333333	0.013461909	0.280367192
Rnf213	-3.523333333	0.013525357	0.280499128
Sympk	-3.97	0.013536232	0.280499128
Slc39a14	2.253333333	0.013542569	0.280499128
Adgrl1	-3.206666667	0.013573431	0.280499128

Tbx2	-1.466666667	0.013580089	0.280499128
Ssh1	-1.556666667	0.013595523	0.280499128
Wdr81	-1.953333333	0.013608738	0.280499128
Myb	-1.582	0.013621292	0.280499128
Cherp	-3.99	0.013671249	0.280749637
Plcg1	-5.02	0.013671753	0.280749637
Spen	-2.46	0.01370211	0.280979491
Col6a3	-7.603333333	0.013728857	0.281091013
Jup	-1.304	0.013754269	0.281091013
Zdhhc8	-1.6	0.013765063	0.281091013
Zfhx4	-1.023333333	0.013822168	0.28125699
Slc35a2	-2.583333333	0.013855419	0.28125699
Tubd1	-1.203333333	0.013857237	0.28125699
Stat5b	-7.11	0.013867308	0.28125699
Adipoq	-5.987333333	0.013904039	0.28125699
Slx4	-2.06	0.013928776	0.28125699
Syvn1	-6.52	0.013934377	0.28125699
Cyp4f37	-1.23	0.013980847	0.28125699
Ctnnb1	-2.189333333	0.013988659	0.28125699
Scarf2	-3.85	0.014015896	0.28125699
Rarres1	-3.065	0.014018127	0.28125699
Pik3r4	-1.956666667	0.014029735	0.28125699
Dkk3	-7.123333333	0.014040346	0.28125699
Aip	-4.41	0.014090009	0.28125699
Foxo1	-3.393333333	0.014091202	0.28125699
Abcg3l4	-4.706666667	0.014091483	0.28125699
Hspg2	-4.73	0.014099297	0.28125699
C1h11orf58	8.823333333	0.014171187	0.281863785
H3f3aps3	1.546666667	0.014186498	0.281863785
Sash1	-3.8	0.014206225	0.281863785
Abcg3l4	-4.503333333	0.014206611	0.281863785
Hcar2	-5.896666667	0.014231422	0.28189075
Siglec5	-1.315	0.014280742	0.28189075
Adap2	-2.193333333	0.014283585	0.28189075
Ackr4	-4.01	0.014319034	0.28189075
Kirrel1	-2.393333333	0.014351795	0.28189075
Porcn	-2.076666667	0.014371633	0.28189075
Ebf3	-5.203333333	0.014372277	0.28189075
Slc52a2	-1.383333333	0.014376827	0.28189075
Snx30	-2.043333333	0.01438209	0.28189075
Pgap1	-1.546666667	0.014431235	0.28189075
Kif5b	3.286666667	0.014444577	0.28189075
Cacna1g	-1.006666667	0.014455205	0.28189075
Ms4a6bl1	-3.44	0.014457908	0.28189075

Golga2	-3.64	0.014487653	0.282095571
Cdh1	-1.051	0.014510647	0.282168581
Rnf26	-3.286666667	0.014542774	0.282418742
Runx1	-1.086333333	0.014574006	0.282558659
Tfb1m	-1.48	0.014613081	0.282558659
Gp5	-7.43	0.014619806	0.282558659
Dcakd	-1.963333333	0.014627065	0.282558659
Abcc10	-1.15	0.014686988	0.283042889
Sirpa	-1.607	0.014725247	0.283042889
Ankrd52	-2.76	0.014746195	0.283042889
Flot2	7.983333333	0.014747117	0.283042889
Pdlim5	-2.843666667	0.014748654	0.283042889
Polr1a	-1.403333333	0.014789207	0.283342828
Galnt11	-1.696666667	0.014802933	0.283342828
Lilra5	-5.73	0.014888942	0.284617568
Nuak1	-1.833333333	0.014940919	0.285104524
Mmp24os	-3.246666667	0.01497731	0.285104524
Abcg1	-6.603333333	0.014987255	0.285104524
Rhbdf2	-1.067666667	0.014995983	0.285104524
Efnb1	-4.366666667	0.015011642	0.285104524
Lix1l	-7.443333333	0.015037185	0.285220186
Lhpp	-1.866666667	0.015139649	0.28679267
Col8a2	-2.266666667	0.01524633	0.288111552
Bmp1	-7.496666667	0.015248572	0.288111552
Abcc4	-1.17	0.015375397	0.290133941
Lrrk1	-1.973333333	0.015440146	0.290686283
Chchd3	1.461	0.01544432	0.290686283
Esm1	-5.166666667	0.015500509	0.291158892
Sema3d	-2.873333333	0.015521614	0.291158892
Dynll1	-1.643666667	0.015539617	0.291158892
Mxi1	1.588666667	0.015548862	0.291158892
Smarca2	-3.88	0.015582726	0.291294074
Trappc13	4.72	0.015619269	0.291360975
Shtn1	-1.22	0.015666508	0.291593351
Bicd2	-4.586666667	0.01569587	0.291593351
Ehmt2	-6.366666667	0.015706601	0.291593351
Ddx31	-1.23	0.015711645	0.291593351
Tcf4	-3.306666667	0.01573909	0.291593351
Adgrl2	-2.79	0.015751053	0.291593351
Hpgds	-7.633333333	0.015793632	0.292012911
Ncdn	-1.966666667	0.015815008	0.292039865
Pdzd2	-1.536666667	0.015858344	0.292060736
Slit3	-2.59	0.015869947	0.292060736
Phc3	-1.8	0.015875897	0.292060736

Smpd1	-1.003333333	0.015940385	0.292339612
Socs3	1.182	0.015965829	0.292339612
Rin3	-7.243333333	0.015968119	0.292339612
Rhobtb2	-1.13	0.015970811	0.292339612
Maf	-5.423333333	0.016009639	0.292390449
Fhip1b	-1.606666667	0.016013472	0.292390449
Poglut3	-2.983333333	0.016037693	0.292468471
Kctd17	-1.176666667	0.016096534	0.293176864
Ttll1	-1.083333333	0.016171388	0.294163273
Arhgef1	-1.548	0.016190817	0.294163273
Bmp3	-1.82	0.016230421	0.294517859
Mrrf	-1.67	0.016271524	0.294898743
Atf6b	-8.186666667	0.016294435	0.294944995
Supt6h	-4.433333333	0.016316791	0.294944995
Spic	-5.7	0.016372826	0.294944995
Tmem259	-5.56	0.016374657	0.294944995
Gtf3c1	-3.056666667	0.016403167	0.295095989
Wrn	-1.533333333	0.016465335	0.295101223
Ednra	-1.92	0.016514157	0.295101223
Pdcd4	-3.951666667	0.016519389	0.295101223
C9h6orf132	-1.07	0.016524219	0.295101223
Ell3	-3.69	0.016626766	0.295832639
Afdn	-6.426666667	0.016646731	0.295832639
Map3k15	1.125333333	0.016657797	0.295832639
Kremen1	-2.86	0.016718291	0.295832639
Dnmbp	-1.043333333	0.016722926	0.295832639
Lrp1	-1.208666667	0.016723083	0.295832639
Rmnd5a	3.24	0.01672659	0.295832639
Cops2	5.926666667	0.016758224	0.296035042
Niban3	-1.345666667	0.016780751	0.296076253
Arl13b	-1.503333333	0.016834825	0.296673319
Cep250	-1.83	0.016864056	0.296831674
Dld	8.88	0.01690357	0.297012316
Sh3rf1	-1.206666667	0.016914833	0.297012316
Ankrd13d	-1.576666667	0.017025073	0.297121703
Nr2f2	-3.48	0.017043108	0.297121703
Olfml2a	-5.96	0.017066008	0.297121703
Cacnb3	-1.176666667	0.017129317	0.297121703
Wdr6	-2.143333333	0.017147955	0.297121703
Rnf144a	-1.51	0.01718371	0.297121703
Tril	-1.076666667	0.017187607	0.297121703
Tsc2	-1.896666667	0.017191746	0.297121703
Gpr34	-3.3	0.017192417	0.297121703
Gdf11	-1.226666667	0.017261136	0.297121703

Dipk1b	-1.103333333	0.017265468	0.297121703
Sfxn3	-5.886666667	0.017283597	0.297121703
Prrt2	-1.403333333	0.01732336	0.297121703
Mob2	4.074666667	0.017324323	0.297121703
Smad9	-1.1	0.017362158	0.297121703
Pofut2	-2.126666667	0.017368866	0.297121703
Kifc3	-1.963333333	0.017380986	0.297121703
Samd9l	-7.006666667	0.017401641	0.297121703
Sipa1l3	-1.7	0.01741655	0.297121703
Capn5	-3.876666667	0.017427606	0.297121703
Mansc1	-1.143333333	0.017428456	0.297121703
Chtf8	-7.303333333	0.017460667	0.297121703
Gpr18	-1.726666667	0.017467757	0.297121703
Setx	-2.953333333	0.017470236	0.297121703
Ift172	-1.336666667	0.017511308	0.297121703
Adgre1	-7.716666667	0.017516422	0.297121703
Ap4s1	1.553333333	0.017567571	0.297121703
Nfia	-1.516666667	0.017570645	0.297121703
Mrc2	-5.543333333	0.017573612	0.297121703
Ccdc93	-1.596666667	0.017607449	0.297121703
Fam78b	-1.29	0.017645704	0.297121703
Pbx1	-1.566666667	0.017652402	0.297121703
Akap17b	-1.006666667	0.017661361	0.297121703
Sytl2	-2.136666667	0.017695782	0.297121703
Tcf7	-1.526666667	0.017699978	0.297121703
Plin1	-1.485666667	0.017733056	0.297121703
Abcc5	-5.47	0.017778982	0.297121703
Scn7a	-1.246666667	0.017819819	0.297121703
Skp1	3.083333333	0.017841854	0.297121703
Abhd14b	-2.536666667	0.017845144	0.297121703
Evl	-1.093	0.017855376	0.297121703
Cd6	-1.143333333	0.017857024	0.297121703
Rab40b	-1.066666667	0.017897781	0.297121703
Lztr1	-3.443333333	0.017907486	0.297121703
Maml1	-1.806666667	0.017928055	0.297121703
Shprh	-1.156666667	0.017940025	0.297121703
Chmp4c	-2.333333333	0.017963913	0.297121703
Sec14l5	-1.226666667	0.017965771	0.297121703
Adora1	-1.46	0.018018177	0.297121703
Isyna1	-9.856666667	0.018029908	0.297121703
Edc4	-7.826666667	0.018143987	0.297121703
N4bp2	-1.4	0.018154808	0.297121703
Mx1	-7.693333333	0.018171957	0.297121703
Sesn3	-4.39	0.018198192	0.297121703

Phactr4	-1.826666667	0.018204897	0.297121703
Tap2	-1.023666667	0.01821178	0.297121703
Slc7a5	9.24	0.018226931	0.297121703
Hdac9	-2.096666667	0.018230261	0.297121703
Glb1l	-1.843333333	0.0182683	0.297121703
Dact1	-1.76	0.018306671	0.297121703
Csf1	-5.846666667	0.018323398	0.297121703
Stard13	-1.02	0.018358228	0.297121703
Neurl4	-2.053333333	0.018366159	0.297121703
Lrp5	-3.233333333	0.018388968	0.297121703
Rasa3	-4.747666667	0.018413804	0.297121703
Tmem106a	-2.01	0.01841411	0.297121703
Cd180	-3.686666667	0.018441624	0.297121703
Fam193a	-1.68	0.018455181	0.297121703
Xaf1	-7.91	0.018460301	0.297121703
Pdgfa	-3.65	0.018491544	0.297121703
Syne2	-3.893333333	0.018493251	0.297121703
Fam110a	-1.503333333	0.018580515	0.297121703
Acsf3	-1.566666667	0.01859608	0.297121703
Fbxl19	-1.833333333	0.018621657	0.297121703
Zbtb7c	-1.43	0.018637009	0.297121703
Wwtr1	-3.016666667	0.018704503	0.297121703
Pxdc1	-5.373333333	0.018743198	0.297121703
Slc27a1	-5.69	0.018749701	0.297121703
Gpt	-3.53	0.018756168	0.297121703
Bace1	-1.52	0.0187592	0.297121703
Inf2	-1.307666667	0.018767069	0.297121703
Zc3h7b	-3.74	0.018772834	0.297121703
Oasl2	-1.189333333	0.01877766	0.297121703
Car8	2.33	0.018808999	0.297121703
Mfsd12	-3.876666667	0.01881883	0.297121703
Cbr1	-2.123333333	0.018848676	0.297121703
Mmp23	-1.266666667	0.018871797	0.297121703
Phf8	-2.246666667	0.018883335	0.297121703
Iffo2	-2.516666667	0.018922203	0.297121703
Pigs	-3.206666667	0.018988987	0.297121703
Ier2	-2.906	0.019026499	0.297121703
Tiam2	-1.17	0.01903087	0.297121703
Acsf2	-1.946666667	0.019049121	0.297121703
Lonp2	-2.67	0.019051812	0.297121703
Col4a2	-9.273333333	0.019101453	0.297121703
Sgpp2	-2.3	0.019155176	0.297121703
Pmepa1	-5.376666667	0.019157708	0.297121703
Gmcl1	-1.953333333	0.019164915	0.297121703

C13h1orf115	-5.676666667	0.019176596	0.297121703
Tubgcp6	-1.423333333	0.019181822	0.297121703
Vps13c	-1.02	0.019199531	0.297121703
C20h6orf89	3.39	0.019242174	0.297121703
Septin8	-7.273333333	0.019245422	0.297121703
Ccdc71	-1.963333333	0.019245545	0.297121703
Tex2	-2.913333333	0.019246405	0.297121703
Bok	-2.053333333	0.019251793	0.297121703
Mtmr11	-2.783333333	0.019301114	0.297121703
Cd86	3.366666667	0.019312303	0.297121703
Tmem268	2.636666667	0.019359204	0.297531076
Fzd7	-1.896666667	0.019425042	0.298078745
Tfrc	19.57533333	0.019453597	0.298078745
Dennd2b	-2.65	0.019489962	0.298078745
Tcf3	-1.415333333	0.019500431	0.298078745
Cdh23	-1.786666667	0.019516796	0.298078745
Ash1l	-2	0.019516819	0.298078745
Tanc1	-2.173333333	0.019611352	0.298763176
Irf8	-1.667666667	0.019621779	0.298763176
Ptcd1	-1.36	0.019622762	0.298763176
Cebpg	4.95	0.019658487	0.298851
Tp53	-1.179	0.019669296	0.298851
Tex264	-1.035333333	0.019719343	0.299057338
Ano1	-1.753333333	0.01972367	0.299057338
Tfpt	2.83	0.019780057	0.299431971
Plxna2	-1.266666667	0.019789222	0.299431971
Hspbp1	-2.35	0.019879447	0.299625677
Dtx3	-6.896666667	0.019883766	0.299625677
Kmt2e	-6.26	0.019914142	0.299775312
Lgi4	-1.986666667	0.01996914	0.300201611
Hoxd8	-2.313333333	0.019992282	0.300201611
Trmt10b	-1.103333333	0.020003886	0.300201611
Mex3d	-1.08	0.020089779	0.301106148
Dgkz	-1.870666667	0.020111505	0.301106148
Kctd15	-3.763333333	0.020142031	0.301106148
Sigmar1	-1.118333333	0.020146306	0.301106148
Itga2b	-1.866	0.020227027	0.301488643
Slc28a2	5.227	0.020239827	0.301488643
Cfap298	-3.61	0.020244214	0.301488643
Reln	-1.303333333	0.020270292	0.301488643
Pkhd1l1	-1.083333333	0.02027471	0.301488643
Nckap5l	-1.416666667	0.020306723	0.301658734
Utp20	-1.386666667	0.02033692	0.301801533
Tapbp	-1.199333333	0.020377208	0.301997141

Cab39l	1.015333333	0.020391295	0.301997141
Ptpn11	-2.853333333	0.020454608	0.302024555
Mdfic	-3.533333333	0.020496067	0.302024555
Otud1	-6.44	0.020510656	0.302024555
Dnpep	-2.563333333	0.02051601	0.302024555
Arhgap39	-1.533333333	0.020533592	0.302024555
Usp18	-6.946666667	0.02053734	0.302024555
Pcdh7	-2.716666667	0.020579407	0.302339946
Exd2	-1.023333333	0.020626842	0.302733489
Ptprv	-1.233333333	0.020694166	0.303417859
Nuak2	6.523333333	0.020756085	0.303549715
Slc7a8	-2.943333333	0.020765268	0.303549715
Syk	-1.812333333	0.020848571	0.30416095
Rpp14	2.796666667	0.020977689	0.304864719
Tinagl1	1.539333333	0.020997213	0.304864719
Ybx1ps20	2.949333333	0.020997475	0.304864719
Aif1l	-1.383333333	0.021000775	0.304864719
Megf8	-1.583333333	0.02103182	0.304961639
Ctnnd1	-1.724	0.021096957	0.304961639
Apol7a11	-3.75	0.021102227	0.304961639
Lig3	-2.16	0.021149238	0.304961639
Mfap3l	-1.876666667	0.021165956	0.304961639
AABR07043951.1	1.11	0.021215996	0.304961639
Ltbp3	-5.75	0.021331508	0.304961639
Abr	-4.29	0.02134403	0.304961639
Nlgn2	-1.306666667	0.02134599	0.304961639
Plekhg1	-1.04	0.021397253	0.304961639
Ubr2	-4.106666667	0.021405644	0.304961639
Zmym6	-1.77	0.02141179	0.304961639
Plxnb2	-1.195	0.021412262	0.304961639
Tnc	-1.200333333	0.021413316	0.304961639
Srsf9	-6.286666667	0.021463436	0.304961639
Top1mt	-1.44	0.021465297	0.304961639
Xkr8	-1.206666667	0.021482407	0.304961639
Pla2g15	-9.926666667	0.02150672	0.304961639
Tp53bp1	-1.266666667	0.02157866	0.304961639
Mroh1	-1.49	0.021587093	0.304961639
Telo2	-1.16	0.021596926	0.304961639
Mknk1	5.304333333	0.021649649	0.304961639
Rnf166	8.373333333	0.02166021	0.304961639
Sned1	-2.87	0.021660595	0.304961639
Tm4sf1	-1.371	0.021661169	0.304961639
Kcnj2	-1.253333333	0.021664947	0.304961639
Miga2	-3.226666667	0.02170335	0.305085795

Etfbkmt	4.396666667	0.021723474	0.305085795
Frmd8	-3.206666667	0.021746992	0.305123831
S1pr3	1.38	0.021805989	0.305374372
Bmf	-2.673333333	0.021806504	0.305374372
Slit2	-3.653333333	0.021945651	0.306389685
Sf3a2	-1.809333333	0.021956258	0.306389685
Jag1	-1.526666667	0.021960332	0.306389685
Pde8a	-1.966666667	0.021962594	0.306389685
Cnnm3	-1.626666667	0.021998883	0.3066042
Abhd17c	-1.836666667	0.02203119	0.306762878
Pik3r2	-3.31	0.022059949	0.30687189
Fzd6	-2.056666667	0.022094632	0.307003147
Spi1	-7.96	0.022113077	0.307003147
Rpusd1	-1.563333333	0.022140227	0.307003147
Scpep1	-3.318	0.022153468	0.307003147
Orai1	1.801666667	0.022174078	0.307003147
Mx2	-1.722	0.022223047	0.307058682
Ndst1	-1.36	0.022223547	0.307058682
Maip1	2.566666667	0.022249755	0.307058682
Fos	-3.655666667	0.022266795	0.307058682
Agrn	-2.166666667	0.022314906	0.307058682
Lin37	-2.743333333	0.022316782	0.307058682
Klhl42	-1.67	0.022327785	0.307058682
Ddr2	-4.686666667	0.022350055	0.307058682
Kdm4b	-1.066666667	0.022375957	0.307058682
Tbc1d13	-1.043333333	0.022405132	0.307058682
Plvap	-1.759333333	0.022408457	0.307058682
Cystm1	1.399	0.022460973	0.30749093
LOC102547042	-1.2	0.022489906	0.307540265
Gpr88	-1.43	0.022509814	0.307540265
Pnpla6	-1.373333333	0.022529461	0.307540265
Rufy4	-1.033333333	0.022568835	0.307540265
Bcl6b	-1.2	0.022570143	0.307540265
Lrrc8a	-1.42	0.022592394	0.307540265
Ptprf	-4.743333333	0.02266167	0.307674303
Gpatch2	-1.236666667	0.022684941	0.307674303
Fhod1	-7.486666667	0.022688671	0.307674303
Pusl1	-2.64	0.022705197	0.307674303
Hey1	-1.213333333	0.022820953	0.308564346
Tcf7l2	-2.246666667	0.02282244	0.308564346
Mcf2l	-1.706666667	0.022866239	0.308564346
Ccz1	1.186333333	0.022868225	0.308564346
Socs2	3.356666667	0.022876104	0.308564346
Nat10	-2.136666667	0.022945037	0.309103842

Ndufa4l2	-1.060666667	0.022975493	0.309103842
Egfl6	-1.246666667	0.022979347	0.309103842
Zfta	-1.963333333	0.02301305	0.309273448
Snd1	-7.763333333	0.023037304	0.309293573
Pdgfc	-2.946666667	0.023056737	0.309293573
Hexa	-1.169333333	0.023113631	0.309633845
Notch3	-4.333333333	0.023124339	0.309633845
Mtarc2	-5.366666667	0.023229393	0.310393507
Abcb8	-1.56	0.02325035	0.310393507
Sh3bp4	-1.483333333	0.023256138	0.310393507
Rusf1	-3.52	0.023370256	0.31108964
Irak1bp1	-1.4	0.023381584	0.31108964
Elf4	-1.936666667	0.023433778	0.31140703
Plekhh3	-2.856666667	0.023448573	0.31140703
Snx17	-4.816666667	0.023469156	0.31140703
Cbx6	-3.68	0.023532301	0.311962565
ST7	3.096666667	0.023582525	0.312099248
Tmem263	-3.35	0.023585184	0.312099248
Helz2	-3.05	0.02363766	0.312511608
Cited4	-1.281333333	0.023663988	0.312577829
Slc22a17	-1.89	0.023717545	0.312748683
Lasp1	-1.358333333	0.023765842	0.312748683
Prkag2	-1.396666667	0.023772441	0.312748683
Copa	-9.033333333	0.023783575	0.312748683
Eng	-7.746666667	0.023805173	0.31275219
Por	-7.763333333	0.02383294	0.312767548
Nfkbia	3.364	0.023932276	0.312767548
Ylpm1	-2.22	0.02398345	0.312767548
Med6	5.093333333	0.0240046	0.312767548
Gnptab	-2.776666667	0.024027657	0.312767548
Sirt5	-6.866666667	0.024029654	0.312767548
Aasdh	-2.093333333	0.024030086	0.312767548
Golgb1	-1.463333333	0.024031316	0.312767548
Vars2	-1.346666667	0.024038859	0.312767548
Rspo3	-1.586666667	0.024069512	0.312767548
Tmem179	-1.27	0.024121237	0.312767548
Aplf	-8.41	0.0241271	0.312767548
Bglap	-1.097406667	0.024132089	0.312767548
Fchsd1	-2.12	0.024155082	0.312767548
Rin2	-8.116666667	0.024171185	0.312767548
Sclt1	8.566666667	0.024173173	0.312767548
Kbtbd7	5.72	0.024174245	0.312767548
Cd72	-2.223666667	0.024210948	0.312767548
Cdc42bpg	-2.393333333	0.02426506	0.312767548

Cnksr3	-1.916666667	0.024265098	0.312767548
Copz1	9.493333333	0.024268242	0.312767548
Pias4	-1.28	0.024275642	0.312767548
Afap1l2	-2.273333333	0.02433623	0.313058378
Csk	-1.928333333	0.024340919	0.313058378
RT1M2	-2.033333333	0.024472922	0.314204886
Srgap3	-1.283333333	0.024564217	0.31470375
Zfp619l1	2.253333333	0.024597395	0.31470375
Rcbtb2	-3.703333333	0.024603434	0.31470375
Dhx37	-1.886666667	0.024619097	0.31470375
Pcdh18	-2.103333333	0.024675123	0.315071928
Mest	-3.72	0.024711233	0.315071928
Mlxip	-1.656666667	0.024722538	0.315071928
Xpo5	-3.046666667	0.024760619	0.315071928
Bbox1	-1.92	0.024777593	0.315071928
Armcx1	-1.553333333	0.0248303	0.315071928
Kmt2b	-2.086666667	0.024838286	0.315071928
Sec16b	-1.09	0.024841301	0.315071928
Ankdd1b	5.25	0.024962834	0.316339729
Nck2	-8.973333333	0.025025224	0.316678804
Cat	-8.633333333	0.025032788	0.316678804
Vps13d	-1.746666667	0.025076473	0.316957972
Zfyve16	-1.033333333	0.025170995	0.317872735
Pdxk	-1.293333333	0.025192206	0.317872735
Arl3	-7.373333333	0.025286431	0.318454042
Mtr	-1.143333333	0.025295754	0.318454042
Sema4c	-1.686666667	0.025303435	0.318454042
Xk	-1.613333333	0.025371881	0.318495303
Cyp4f39	-1.173333333	0.025448942	0.319189378
Msmo1	-9.75	0.025579539	0.320331482
Ushbp1	-1.666666667	0.025643959	0.320812057
Zfp395	-2.3	0.025683785	0.320962427
Dpep3	-1.02	0.025772745	0.321419678
Mtor	-1.573333333	0.025782295	0.321419678
Parp14	-7.166666667	0.025843598	0.321419678
Akap11	-1.256666667	0.025843824	0.321419678
Tlcd3a	-3.113333333	0.025845983	0.321419678
Ufl1	2.383333333	0.025908001	0.321861557
Evi2a	-1.269666667	0.025970799	0.321861557
Hid1	-1.71	0.026019899	0.321861557
Sncg	-4.419	0.026023039	0.321861557
Cdan1	-2.266666667	0.026035001	0.321861557
Rcn3	-1.961	0.026035074	0.321861557
Arpc5l	-4.008666667	0.026036009	0.321861557

Mthfd2	2.416333333	0.026081989	0.321861557
Gon4l	-1.483333333	0.026085816	0.321861557
Sbk1	-2.123333333	0.026137845	0.321861557
Or6k2	-1.226666667	0.026167208	0.321861557
Tgfbr2	-1.439666667	0.026185964	0.321861557
Smpd3	-1.012	0.026201135	0.321861557
Tyk2	-3.106666667	0.026210797	0.321861557
Rmnd5b	-2.55	0.026235881	0.321899992
Rftn2	-1.683333333	0.026378632	0.323380858
Arhgef40	-1.833333333	0.026412038	0.323519879
Tyro3	-1.683333333	0.026511159	0.324013339
Nek8	-1.06	0.02651862	0.324013339
Dpysl3	-3.716666667	0.026572231	0.324398044
Icam1	-3.266666667	0.026632317	0.32486109
Ikzf5	4.02	0.026654579	0.324862372
Atp6ap1l	1.588666667	0.026728991	0.325166459
Calhm5	-1.146666667	0.026763516	0.325166459
Tmtc4	-1.213333333	0.026844513	0.325166459
Col18a1	-4.253333333	0.0268498	0.325166459
Slc4a1	7.285333333	0.026857758	0.325166459
Septin4	-6	0.026894253	0.325166459
Kdelr3	-7.86	0.02690477	0.325166459
Mdfi	-1.786666667	0.026918974	0.325166459
Lgr4	-2.493333333	0.027021675	0.326038847
Sipa1l2	1.186666667	0.0270579	0.326038847
Gapdh	1.81582	0.02706562	0.326038847
Wdr46	-2.356666667	0.027084662	0.326038847
Mus81	-1.233333333	0.027144122	0.326342684
Rps9l1	3.866666667	0.027302512	0.327853752
Bmp4	-5.186666667	0.027383529	0.328557534
Tmem255b	-1.38	0.027473895	0.329372237
Etfb	8.020666667	0.027559707	0.329980831
Mtm1	-1.66	0.0275893	0.329980831
Epas1	-1.102333333	0.027603094	0.329980831
Bambi	-7.79	0.027614683	0.329980831
Adcy9	-1.233333333	0.027704807	0.330594683
Fkbp5	2.809666667	0.027737166	0.330594683
Aox1	-1.713333333	0.027749193	0.330594683
Thumpd2	-1.146666667	0.027760695	0.330594683
Pard3	-1.896666667	0.027778907	0.330594683
Mmp2	-3.089666667	0.02780134	0.330594683
Pou2f2	-2.35	0.027909536	0.331343824
Nme3	-3.276666667	0.027992482	0.332059684
Mdk	-2.68	0.028050015	0.332374801

Rai14	-3.49	0.028077371	0.332374801
Ubl3	7.99	0.028087054	0.332374801
Ccdc28a	4.07	0.028226169	0.332644537
Cadm1	-5.646666667	0.028229753	0.332644537
Slc7a15	-1.49	0.028236847	0.332644537
Nradd	-1.01	0.028294743	0.332644537
Sox13	-1.256666667	0.028298423	0.332644537
Atxn2	-2.243333333	0.028364346	0.332644537
L3mbtl3	-2.44	0.028384702	0.332644537
Mib2	-1.27	0.028401809	0.332644537
Fbln5	-2.426666667	0.028404785	0.332644537
Socs1	1.493333333	0.02857454	0.334098812
Fam13c	-1.403333333	0.028655002	0.334772616
Itpr2	-1.48	0.028774258	0.335115547
Gpc6	-1.2	0.02881404	0.335115547
Tmem204	-2.166666667	0.028822881	0.335115547
Fbxo4	-1.556666667	0.028839526	0.335115547
Fam120a	-9.62	0.028840735	0.335115547
Slc3a2	1.428333333	0.028842567	0.335115547
Glg1	-4.196666667	0.028851632	0.335115547
Sh3glb2	-3.416666667	0.028884254	0.335115547
Gp1ba	-1.706666667	0.028923841	0.335119424
Fam149a	-1.073333333	0.028936106	0.335119424
Col4a1	-1.129	0.029071823	0.335641983
Birc3	2.453333333	0.029088844	0.335641983
Lrrc75b	-1.483333333	0.029094234	0.335641983
Dhx58	-4.26	0.029095687	0.335641983
Mxra8	-2.336666667	0.029123516	0.335655023
Tars3	8.26	0.029142603	0.335655023
Naprt	-7.796666667	0.029195672	0.335951541
Pacc1	-1.54	0.029235937	0.335951541
Slc25a21	5.176666667	0.029237087	0.335951541
Ptgir	-4.043333333	0.029312486	0.336255164
Slc38a1	-7.996666667	0.029332312	0.336255164
Map1b	-1.303333333	0.02936097	0.336320741
Slc10a6	1.16	0.029389924	0.336389589
Zfp202	-2.383333333	0.029432487	0.33640016
LOC100909928	-2.19	0.029486892	0.33640016
Slc38a5	1.93	0.029512089	0.33640016
Plekha3	1.858333333	0.029512471	0.33640016
Pnpla3	-1.29	0.02951735	0.33640016
Smg7	-2.47	0.029595032	0.33640016
Nif3l1	-2.093333333	0.02963479	0.33640016
Plekhg5	-2.066666667	0.029647039	0.33640016

Prpf8	-8.413333333	0.029664509	0.33640016
Pcdh1	-1.306666667	0.029673828	0.33640016
Phaf1	1.886666667	0.029695289	0.33640016
Mamdc2	-3.313333333	0.029707058	0.33640016
Rtkn	-1.443333333	0.029712059	0.33640016
Acss1	-2.8	0.02976714	0.336566435
Ccnd1	-5.413333333	0.029790421	0.336566435
Jpt1	-7.693333333	0.0298183	0.336566435
Pole4	8.613333333	0.029818565	0.336566435
Hexb	-3.893333333	0.029955013	0.337512654
Ift43	-2.25	0.029980061	0.337512654
Mbtps1	-3.633333333	0.029999511	0.337512654
Clstn1	-5.68	0.030017506	0.337512654
Cops3	4.08	0.030024127	0.337512654
Xkrx	-1.253333333	0.030040514	0.337512654
Aqp7	-2.573333333	0.030080689	0.337705251
Fndc3b	-2.38	0.030171237	0.338462647
Sat1	1.0855	0.030234537	0.3388169
Atp1b2	-1.199	0.030249033	0.3388169
Wrnip1	-7.473333333	0.030352669	0.339485844
Mical1	-8.496666667	0.030388099	0.339596269
Gbf1	-2.613333333	0.030425574	0.339756106
Shank3	-1.903333333	0.030505721	0.340391842
Cdk5	-1.216666667	0.030559019	0.34072725
Fam98a	-3.28	0.030638717	0.341320317
Sar1b	-6.541	0.030658768	0.341320317
Lrrc75a	-1.313333333	0.030725436	0.341607954
LOC120101644	9.913333333	0.030731202	0.341607954
Marchf1	9.006666667	0.03084298	0.341637108
Sh3pxd2b	-1.756666667	0.030877858	0.341637108
Abcc3	-1.773333333	0.030891735	0.341637108
Rell1	4.703333333	0.030896492	0.341637108
Ier5	-6.383333333	0.030954148	0.341637108
Hacd4	-4.403333333	0.030974342	0.341637108
Zfp219	-3.41	0.030981019	0.341637108
Rasal1	-1.666666667	0.031100686	0.341637108
Itpr1	-1.416666667	0.031111703	0.341637108
Golim4	-6.82	0.031117646	0.341637108
Tlr2	1.346	0.031120124	0.341637108
Adh5	5.103333333	0.031138103	0.341637108
Mak	2.486666667	0.031149761	0.341637108
F2rl2	4.536666667	0.031153915	0.341637108
Tmem132a	-3.926666667	0.031216163	0.341637108
Akirin2	3.953333333	0.031305348	0.341637108

Septin5	-1.331333333	0.031309463	0.341637108
Ubfd1	-1.926666667	0.03131292	0.341637108
Caskin2	-1.756666667	0.031328638	0.341637108
Atp6v0b	8.36	0.031330187	0.341637108
Gga2	-6.843333333	0.031341146	0.341637108
Vash1	-1.32	0.031348947	0.341637108
Umps	-6.55	0.031373032	0.341637108
Cdc42bpb	-2.04	0.031378034	0.341637108
Prdx3	2.597666667	0.031454352	0.341995682
Dop1b	-1.086666667	0.031465842	0.341995682
Zdhhc9	-2.396666667	0.031521357	0.342125677
Hdac5	-1.434666667	0.031524471	0.342125677
Lst1	4.810666667	0.031563023	0.342290714
Rasl12	-1.6	0.031612151	0.342529008
Dgkq	-1.303333333	0.03163172	0.342529008
Nr4a3	3.826666667	0.031657483	0.342554994
Atp6v1d	7.19	0.031698458	0.342745424
Tmem170b	2.523333333	0.031735988	0.342898341
Pwp2	-1.923333333	0.031802827	0.342968265
Mon2	-1.283333333	0.031804656	0.342968265
Nipal3	-1.753333333	0.031812634	0.342968265
Arhgef11	-1.78	0.031865125	0.343281756
Trim14	-1.143333333	0.031927699	0.343703325
Zc3h13	-9.373333333	0.032011868	0.344356578
Gpr84	-1.326666667	0.032117152	0.34443011
Nbeal2	-1.163333333	0.032122247	0.34443011
Camkk1	-2.306666667	0.032128525	0.34443011
Hoxa11	-2.323333333	0.032145214	0.34443011
Slc39a10	-2.553333333	0.032153202	0.34443011
Znrf1	3.856666667	0.032159652	0.34443011
Rpl26ps1	1.42	0.032213512	0.344690097
Atp6v1h	2.83	0.032239203	0.344690097
Os9	-7.746666667	0.032254455	0.344690097
Arl4c	-2.013333333	0.032286739	0.344702568
Fads3	-2.226666667	0.032302641	0.344702568
Ezr	-1.946333333	0.03242274	0.345510415
Tceal8	3.203333333	0.032439419	0.345510415
Ppp1cb	4.203333333	0.032474101	0.345510415
Gna12	-4.503333333	0.0324955	0.345510415
Stap2	-2.763333333	0.032496171	0.345510415
Rilpl1	-8.01	0.032530371	0.345623406
Plxnb1	-1.556666667	0.032670084	0.346352172
Mia3	-3.13	0.032694084	0.346352172
Sema3g	-1.72	0.032750977	0.346352172

Dnajc7	8.043666667	0.032752589	0.346352172
Lss	-4.643333333	0.03278782	0.346352172
Gvin1	-1.636666667	0.032791959	0.346352172
Dapk1	-1.413333333	0.032803999	0.346352172
Kat6a	-3.106666667	0.032811565	0.346352172
Ablim3	-1.756666667	0.032840844	0.34641184
Dtx2	-2.146666667	0.032871924	0.346490404
Zc2hc1a	-1.783333333	0.03299329	0.346810716
Snrpb2	2.074333333	0.033008481	0.346810716
Rps25l3	11.37466667	0.033020581	0.346810716
Stau1	-2.763333333	0.033070791	0.347089431
Neu1	-9.976666667	0.033131745	0.347287001
Tfpi2	2.596666667	0.033136988	0.347287001
Calr	-4.452333333	0.033180135	0.347490809
Epha2	-1.716666667	0.033279778	0.347718331
Col13a1	-6.146666667	0.033296961	0.347718331
Chaf1b	-1.703333333	0.033313228	0.347718331
Stat5a	-9.806666667	0.033345075	0.347718331
Evc	-1.88	0.033375236	0.347718331
Sema3b	-2.523333333	0.033398615	0.347718331
Letmd1	-1.806666667	0.033432307	0.347718331
Cald1	-1.185666667	0.033492001	0.347718331
Phldb2	-1.133333333	0.03349691	0.347718331
Rabl2	-1.286666667	0.033509813	0.347718331
Afap1	-1.383333333	0.033518403	0.347718331
Dapk3	-5.063333333	0.033523304	0.347718331
Chchd4ps1	2.216666667	0.033545445	0.347718331
Yeats2	-1.54	0.033599336	0.347718331
Bsg	-2.843	0.033632349	0.347718331
Rps20ps10	2.326	0.033639214	0.347718331
Nphp1	-1.296666667	0.033657815	0.347718331
Gmppa	-4.256666667	0.033695357	0.347718331
Usp48	-3.923333333	0.033720237	0.347718331
Ep400	-8.5	0.033723603	0.347718331
Tfb2m	1.806666667	0.033760029	0.347849297
Ndufc1	-1.389233333	0.033838593	0.348256335
Prss29ps1	-7.131	0.033847038	0.348256335
Dnaja2	5.906666667	0.033971904	0.348627279
Thbs1	-3.872	0.034043287	0.348627279
Msl2	-2.03	0.034049432	0.348627279
Bcap29	9.443333333	0.034064636	0.348627279
Erc1	-1.236666667	0.034075203	0.348627279
Prrc2a	-1.995	0.034086297	0.348627279
Cand1	-4.85	0.034113781	0.348627279

Sf3b2	-5.9	0.034119574	0.348627279
Man2b1	-7.305666667	0.034120867	0.348627279
Fkbp4	-1.061	0.034154511	0.348728026
Tmem167a	1.569666667	0.034228076	0.349213834
Hcfc2	6.27	0.034249727	0.349213834
Epb41l1	-2.426666667	0.03433363	0.349826043
Niban1	-6.626666667	0.034412267	0.350323734
Mrpl1	4.74	0.034469985	0.350323734
Tomm20	8.954333333	0.034471414	0.350323734
Il1r2	2.481666667	0.034478049	0.350323734
Crbn	1.560333333	0.034518037	0.350487163
Hectd3	-2.19	0.034579133	0.35086453
Cmpk2	-5.423333333	0.034656476	0.351406117
Sdc3	-7.68	0.034755752	0.35204164
Slco2b1	-6.73	0.034775762	0.35204164
RT1T241	1.356666667	0.034815194	0.35204164
Tlr8	-4.89	0.034846198	0.352112305
Tmem183a	8.37	0.034961867	0.352348366
Rras	-5.513333333	0.034969606	0.352348366
Ms4a6b	-4.756666667	0.03500983	0.352348366
Pcbd1	-2.216666667	0.035019799	0.352348366
Zfp28	-1.046666667	0.035028463	0.352348366
Rap2a	-3.05	0.035037779	0.352348366
Syne3	-1.226666667	0.035070526	0.352435952
Sec24d	-6.106666667	0.035161691	0.353086247
Zfp282	-1.046666667	0.035194182	0.353086247
Rcc2	-1.299666667	0.035251705	0.353086247
Etv1	-1.096666667	0.035297833	0.353086247
Calcrl	-3.083333333	0.035334189	0.353086247
Il17rc	-1.85	0.035356036	0.353086247
Pdgfd	-2.073333333	0.035362894	0.353086247
Slc35b2	-3.156666667	0.035447722	0.353086247
Olfm1	-2.663333333	0.035510989	0.353086247
Rpn2	-1.401	0.035514335	0.353086247
Ell2	1.836666667	0.03553766	0.353086247
Ralgdsl2	-3.76	0.035544029	0.353086247
Ap3d1	-4.193333333	0.035556991	0.353086247
Cerk	-6.31	0.035558449	0.353086247
Epm2aip1	-1.086666667	0.035563012	0.353086247
Ppib	-1.3322	0.035592789	0.353086247
Pstk	1.21	0.035778167	0.354070649
Tstd1	-3.096666667	0.035825032	0.354070649
Mier2	-1.513333333	0.035826235	0.354070649
Emc9	-1.19	0.035836915	0.354070649

Zfp185	-1.163333333	0.035885103	0.354174634
Nebl	-1.916666667	0.035895751	0.354174634
Aldh9a1	-6.46	0.035928687	0.354188132
Ankhd1	-2.353333333	0.035945433	0.354188132
Msantd2	-1.836666667	0.036069349	0.354513728
Orai3	-2.08	0.0360714	0.354513728
Hddc3	-1.32	0.036071863	0.354513728
Il7	-1.06	0.036075193	0.354513728
Lck	-2.02	0.036121928	0.354735241
Nr2c2	2.376666667	0.036209653	0.35512102
Uimc1	2.083333333	0.036253266	0.355151358
Pi15	-1.146666667	0.036261191	0.355151358
Zfp592	-1.766666667	0.036332982	0.355483993
Chp2	-4.19	0.036343644	0.355483993
Mylk	-7.016666667	0.03639851	0.355713646
Ints8	-3.466666667	0.036415645	0.355713646
Mark2	-3.34	0.036466414	0.355972411
Ncoa5	-6.83	0.036538313	0.355982818
Dph5	1.733333333	0.036545657	0.355982818
Tafa3	-1.186666667	0.03662697	0.355982818
Fam193b	-1.142333333	0.036646832	0.355982818
Poc5	1.233333333	0.03667988	0.355982818
Slc7a7	-2.34	0.036734325	0.355982818
Galnt16	-1.31	0.036750414	0.355982818
Cyb5b	-1.365666667	0.036755468	0.355982818
Ost4	-2.538933333	0.036758831	0.355982818
Bmp7	-1.64	0.036816656	0.356072434
Zranb1	4.096666667	0.036868076	0.356078747
Myo19	-1.063333333	0.036873296	0.356078747
Trarg1	-7.12	0.036890166	0.356078747
Gnb1l	-1.656666667	0.036977124	0.356255426
Surf6	1.367666667	0.036981364	0.356255426
Phf12	-4.313333333	0.037041693	0.356602296
Prkd2	-4.326666667	0.037071138	0.356651592
Dpp3l1	-4.29	0.0371499	0.357032055
Lin7c	7.62	0.037159386	0.357032055
Snrnp200	-5.256666667	0.037192034	0.357111725
Slc43a1	6.705	0.037264889	0.357337797
Nr2c1	-1.26	0.037305744	0.357337797
Map3k11	-2.63	0.037336478	0.357337797
Tsku	-1.81	0.037348856	0.357337797
Eps8l2	-2.086666667	0.037353689	0.357337797
Urah	8.233333333	0.037364567	0.357337797
Rps12l3	1.753333333	0.037387506	0.357337797

Phlpp1	-1.126666667	0.037410552	0.357337797
Epb41l4b	-1.946666667	0.03752289	0.358006418
Tmcc2	-2.9339	0.037529386	0.358006418
Bcat2	-6.466666667	0.037592076	0.358371277
Zfp668	-1.056666667	0.037710071	0.359262548
Dock9	-2.886666667	0.037747837	0.359388823
Dock6	-1.636666667	0.037776564	0.359428933
Lrp6	-1.94	0.03791568	0.360284967
Tap1	2.121	0.038064043	0.36102344
Txndc5	-1.58	0.038088408	0.36102344
Prnp	-9.19	0.03809233	0.36102344
Nup93	-3.966666667	0.038112972	0.36102344
Baz1b	-6.083333333	0.0381332	0.36102344
Zmiz1	-6.4	0.038198771	0.361104343
F8	-1.993333333	0.038198938	0.361104343
Cog2	-2.326666667	0.038291047	0.361487091
Edc3	5.713333333	0.038400724	0.361957014
Dido1	-5.9	0.038433054	0.361957014
Cyp2u1	-1.136666667	0.038548934	0.36239929
Gabbr1	-2.13	0.038558375	0.36239929
AABR07054614.1	-8.023333333	0.038609576	0.362648046
Cgnl1	-1.346666667	0.038681429	0.362858042
Slc4a2	-3.64	0.038719213	0.362980247
Mllt6	-1.863333333	0.038841945	0.363665772
Mapk8	3.1	0.038924272	0.363938165
Cyp20a1	-3.096666667	0.039002008	0.364233394
Otulin	3.99	0.039060749	0.364233394
Atp8a1	-2.763333333	0.039075106	0.364233394
C7h8orf33	-5.826666667	0.039133922	0.364233394
Dnajb5	-4.96	0.039147783	0.364233394
Neil1	-6.173333333	0.0391554	0.364233394
Igdcc4	-1.223333333	0.039163073	0.364233394
Men1	-7.173333333	0.039196107	0.364233394
Rcan3	-2.17	0.039237664	0.36434619
Brpf3	-1.473333333	0.039267845	0.364395662
Exoc6	4.436666667	0.039348917	0.364610998
P2rx4	1.873666667	0.039367742	0.364610998
Qpctl	-1.44	0.039461167	0.364610998
Map3k1	-2.596666667	0.039489176	0.364610998
C16h19orf44	-1.683333333	0.039527789	0.364610998
Card9	-2.96	0.039531302	0.364610998
Eef1g	12.21533333	0.039578645	0.364610998
Ccdc32	3.346666667	0.039616145	0.364610998
Tars2	-2.94	0.039644302	0.364610998

Chd6	-1.613333333	0.039699337	0.364610998
Dnttip2	3.16	0.039700939	0.364610998
Ap2a2	5.076666667	0.039836347	0.364610998
Setd3	1.144333333	0.039840337	0.364610998
LOC102556096	-2.57	0.039929025	0.364610998
Abcd3	-1.013666667	0.040005795	0.364610998
Nol10	5.47	0.040025688	0.364610998
Psmd13	5.627333333	0.040033449	0.364610998
Ccrl2	1.006666667	0.040038829	0.364610998
Plbd2	-4.196666667	0.040040655	0.364610998
Itga4	-4.126666667	0.040042663	0.364610998
C6	-4.16	0.040048813	0.364610998
Nipa1	-1.2	0.040058552	0.364610998
Gzmk	6.633333333	0.040069719	0.364610998
Dnaaf10	-2.46	0.040139363	0.364610998
Xpc	-1.54	0.040158885	0.364610998
Ism1	-1.826666667	0.040184076	0.364610998
Rita1	-1.473333333	0.04018756	0.364610998
Septin9	-1.212666667	0.04020449	0.364610998
Cdh2	-2.966666667	0.040216248	0.364610998
Catsperg	-1.833333333	0.040216695	0.364610998
Mff	9.693	0.040243906	0.364610998
Znf865	-1.203333333	0.04025348	0.364610998
Pls1	-2.543333333	0.040266076	0.364610998
Tbc1d2b	-8.276666667	0.040272553	0.364610998
Acvrl1	-3.896666667	0.040322213	0.364679866
Tmem184c	1.13	0.040345454	0.364679866
Coq3	1.437	0.040388158	0.364679866
Cdc42ep4	-3.08	0.040392859	0.364679866
Il20rb	-3.936666667	0.040541032	0.365792378
4930426D05Rikl	-1.333333333	0.040663341	0.3666703
Washc1	-1.746666667	0.040760195	0.366864016
Pdgfb	-2.463333333	0.040788907	0.366864016
Haus2	2.453333333	0.040796327	0.366864016
Oas2	-2.29	0.040820529	0.366864016
Dbr1	4.363333333	0.040862191	0.366864016
Map2k3	1.688666667	0.040867699	0.366864016
Ramp2	-4.893333333	0.040876848	0.366864016
Herc6	-3.78	0.040884995	0.366864016
Emc1	-1.86	0.040955378	0.367190424
Cers4	-2.043333333	0.041013562	0.367190424
Tle2	-1.25	0.04105954	0.367190424
Nipal2	-1.906666667	0.041148311	0.367190424
Iqgap3	-2.136666667	0.041184251	0.367190424

Herc1	-2.49	0.041246873	0.367190424
Aplp2	-4.691666667	0.041275817	0.367190424
Rrp7a	-1.736666667	0.041316127	0.367190424
Jrkl	-1.426666667	0.041358405	0.367190424
Ccdc80	-3.766666667	0.041383269	0.367190424
Elac2	-2.486666667	0.041384071	0.367190424
Galk1	-2.226666667	0.041395947	0.367190424
Irs1	-2.246666667	0.041427774	0.367190424
Ccni	1.661666667	0.041449981	0.367190424
F7	1.183333333	0.041469161	0.367190424
Atraid	6.226666667	0.041510717	0.367190424
Il7r	7.213333333	0.04156311	0.367190424
Abhd4	2.89	0.041655353	0.367190424
Zfp846	-1.17	0.041664115	0.367190424
Atp13a1	-5.263333333	0.041685486	0.367190424
Alg8	-2.11	0.041686933	0.367190424
Dcaf12	-6.856666667	0.041717125	0.367190424
Trappc9	-1.776666667	0.041743686	0.367190424
Fdps	-2.191666667	0.041757273	0.367190424
She	-1.406666667	0.04183851	0.367190424
Heatr1	-2.03	0.041853	0.367190424
Smyd5	-1.216666667	0.041876498	0.367190424
Zswim4	-1.036666667	0.041882888	0.367190424
Traf3ip2	-1.67	0.041894958	0.367190424
Prr14l	-1.57	0.04192892	0.367190424
Slc41a1	-1.29	0.041929418	0.367190424
Ssr3	5.95	0.041983792	0.367190424
Ecd	-2.986666667	0.042018108	0.367190424
Ogn	-3.641	0.042039518	0.367190424
Dip2c	-1.143333333	0.042047824	0.367190424
Mepe	-6.574	0.042051802	0.367190424
Mprip	-2.638666667	0.042063289	0.367190424
Iah1	8.28	0.042101253	0.367190424
Sost	-6.152333333	0.042125682	0.367190424
Abi3bp	-6.276666667	0.042148391	0.367190424
Zdhhc20	7.19	0.042149387	0.367190424
Rhod	-1.386666667	0.042184084	0.367190424
Cgref1	-8.59	0.042189784	0.367190424
Galm	-2.386666667	0.042221453	0.367190424
Cnnm4	-1.72	0.042239754	0.367190424
Ska2	2.427333333	0.042260617	0.367190424
Plk2	-2.266666667	0.042307672	0.367190424
Igsf3	-2.036666667	0.042322578	0.367190424
Scrn2	-1.023333333	0.042330325	0.367190424

Ankra2l1	2.38	0.042338666	0.367190424
Mtatp8	-11.68912	0.042348861	0.367190424
Sf3a1	-8.57	0.0424077	0.367483275
Sec24c	-4.193333333	0.042465018	0.367762607
Esyt1	-1.140666667	0.042509089	0.367858648
Oasl	-5.983333333	0.042526286	0.367858648
Ckap4	-2.413	0.04257459	0.368059342
Col5a3	-2.24	0.042724503	0.368839793
Lrrc14	-1.606666667	0.042743327	0.368839793
Me2	2.013666667	0.04275464	0.368839793
Ubl7	-1.853333333	0.042765492	0.368839793
Ccdc85c	-1.943333333	0.042824287	0.369129746
Nol9	-1.92	0.042865155	0.369264924
Rps25l2	-3.835333333	0.042921299	0.369531467
Tpbg	-1.763333333	0.043079895	0.370679233
Pyroxd2	-1.48	0.043118484	0.370793675
H1f2	13.15933333	0.0431659	0.370887262
Raver1	-1.761333333	0.043204125	0.370887262
AABR07053185.1	1.43	0.043205255	0.370887262
Zfp950l2	2.58	0.043256301	0.371108181
Itgbl1	-6.633333333	0.043325255	0.371482389
Sp110	-8.863333333	0.043367309	0.371539479
Acox1	-1.388	0.04339134	0.371539479
Akap17a	-4.496666667	0.043422085	0.371539479
Ak6	6.766666667	0.043458615	0.371539479
Dnmt3a	-1.08	0.043496031	0.371642664
Serpinf1	-8.175	0.043549734	0.371884799
Nacc1	-3.503333333	0.043625268	0.372212376
Mrpl42	9.4	0.043638867	0.372212376
Nup42	1.5	0.043713625	0.372516528
Slc22a3	-1.183333333	0.043725486	0.372516528
Slc25a35	-1.143333333	0.043754596	0.372516528
Map3k14	-1.583333333	0.043776155	0.372516528
Map7	-1.58	0.043802203	0.37252198
Il3ra	1.226666667	0.044000309	0.373773195
Rbm15b	-2.35	0.044038851	0.373883978
Pcyt1a	-1.404333333	0.044200832	0.374691516
Epb41l2	-1.274	0.044213345	0.374691516
Isca1	3.635666667	0.044346782	0.375337332
Capn2	-9.863333333	0.044363634	0.375337332
Rnd2	-1.44	0.044520585	0.375751701
Dync1li2	-2.166666667	0.044527878	0.375751701
Zfp609	-1.553333333	0.044578658	0.375751701
Ring1	-2.543333333	0.044592004	0.375751701

Tor1b	-6.62	0.044728754	0.375881486
Zer1	2.476666667	0.044768917	0.375881486
Tmem47	-3.196666667	0.044774909	0.375881486
Scarb1	-2.002666667	0.044793344	0.375881486
Ttpal	-6.09	0.044832263	0.375881486
Rsl24d1	2.046666667	0.044850097	0.375881486
Npy	-8.435333333	0.044857046	0.375881486
Ankrd13b	-1.363333333	0.044860675	0.375881486
Kmt5b	-9.936666667	0.044921428	0.375881486
Als2cl	-6.86	0.04493212	0.375881486
Nsd1	-3.436666667	0.044979922	0.375881486
Znf48	-1.13	0.045003885	0.375881486
Arl2	-2.516666667	0.045012687	0.375881486
Crebrf	2.296666667	0.04511045	0.376228341
Nxt2	4.606666667	0.045192086	0.376694916
Pwwp2b	-1.393333333	0.045331829	0.377430592
Tet3	-1.493333333	0.04538301	0.377524419
Uhmk1l1	-4.493333333	0.045476507	0.377991238
Pou2af1	-2.187	0.045686935	0.37923713
Per3	1.436666667	0.045729863	0.37923713
Recql4	-1.343333333	0.045795746	0.379568816
Col15a1	-6.306666667	0.045887613	0.37975478
Klhl25	-2.406666667	0.045915276	0.37975478
Myo10	-1.693333333	0.045916563	0.37975478
Mpc2	-4.293333333	0.045975246	0.379768479
Lgals1	-1.566366667	0.046056536	0.38008097
Dock7	-1.033333333	0.04607152	0.38008097
Rag1	-4.12	0.046112745	0.38008097
Itga10	-1.796666667	0.046116768	0.38008097
Lpar6	-1.666666667	0.046208262	0.380556154
Rhbdl2	-1.17	0.046226334	0.380556154
Wnt16	-1.29	0.046325174	0.38093561
Sema5a	-1.98	0.04633831	0.38093561
Card11	-2.96	0.046376351	0.38093561
Bmyc	-1.856666667	0.046418359	0.381018673
Agap1	-1.816666667	0.046464424	0.381018673
Plekhf1	-1.09	0.04652623	0.381156975
Cdc123	2.189	0.046559279	0.381156975
Scap	-6.326666667	0.04661538	0.381219856
LOC100365839	2.19	0.046673165	0.381219856
Htra1	-2.471	0.046709691	0.381219856
Rcor1	-4.033333333	0.046771204	0.381219856
Lrch3	-8.86	0.046773209	0.381219856
Map1s	-1.393333333	0.046780288	0.381219856

F11r	-4.18	0.046802322	0.381219856
Dmwd	-1.343333333	0.046813185	0.381219856
Mtnd5	17.89266667	0.046826965	0.381219856
Clta	13.19533333	0.046927907	0.381662083
Sema3f	-1.893333333	0.046933347	0.381662083
Tmed9	-8.96	0.046995159	0.381952898
Ccdc9	-2.746666667	0.047142493	0.382545646
Tmem238	-2.193333333	0.047178713	0.382545646
Nol6	-1.296666667	0.047196361	0.382545646
Pigv	-2.01	0.047259916	0.382545646
Ppme1	-1.793333333	0.047292922	0.382545646
Cracr2b	-1.193333333	0.047296942	0.382545646
Prkca	-8.38	0.047381565	0.382545646
Arhgap27	-7.693333333	0.047383599	0.382545646
Psmd1	3.321666667	0.047406391	0.382545646
Cttnbp2	-1.06	0.047433411	0.382545646
Adcy5	-1.61	0.047434025	0.382545646
Ighm	-2.686936667	0.047441099	0.382545646
Pdf	-2.033333333	0.047475672	0.382545646
Ccdc187	-1.04	0.047496478	0.382545646
Alas1	-8.966666667	0.047511637	0.382545646
Uba7	-1.773333333	0.047579092	0.38287851
Slc39a13	-4.386666667	0.047672163	0.383417032
Tab3	-5.03	0.047724666	0.383628865
Pomk	-2.096666667	0.047806546	0.383977206
Tmx2	3.58	0.047831908	0.383977206
Tpgs1	-1.43	0.047846566	0.383977206
Ppp1r10	-1.151666667	0.048032569	0.385089074
Tubb6	-1.633333333	0.048043381	0.385089074
Wbp1	5.473333333	0.048099367	0.385162711
Cavin3	-2.047	0.048152223	0.385375492
Ift140	-1.866666667	0.048202548	0.38556779
Ston1	-1.213333333	0.048246552	0.385620066
Kif21b	-1.76	0.048261685	0.385620066
Gamt	-1.693333333	0.048289262	0.385630264
Neo1	-2.81	0.048334155	0.385721714
Ubxn2b	1.566666667	0.048373383	0.385721714
Abhd16a	-4.116666667	0.048420216	0.385721714
Metrnl	-1.496333333	0.048432252	0.385721714
Cpz	-5.2	0.048490775	0.385801994
Dll4	-1.206666667	0.048550995	0.385801994
Mms19	-1.646666667	0.048559229	0.385801994
Dctn5	1.803666667	0.048572415	0.385801994
Etv5	-1.756666667	0.048593172	0.385801994

Smarcb1	-6.25	0.04860021	0.385801994
Ttyh3	-1.043333333	0.048782653	0.386831396
Snx29	-1.73	0.048910036	0.387310101
Cdc42se2	2.471333333	0.048919556	0.387310101
Fbxo8	3.39	0.048922269	0.387310101
Sik2	-2.04	0.04898597	0.387605125
Pnpla8	6.12	0.049058	0.387965697
Hmgxb3	-1.503333333	0.049115396	0.388147519
Tmem88	-1.55	0.049185923	0.388147519
Ckmt1	-2.116666667	0.049263468	0.388147519
Susd2	-1.75	0.049264689	0.388147519
Eif4g3	-4.293333333	0.049318074	0.388147519
Jdp2	1.547	0.04935895	0.388234701
Rasa1	-2.49	0.04939655	0.388234701
Gtf2e1	-1.336666667	0.049443817	0.388246731
Cd93	-1.151666667	0.049486205	0.388246731
Matn2	-1.296666667	0.049492368	0.388246731
Slx1b	-1.87	0.0495347	0.388246731
Nploc4	3.426666667	0.049543693	0.388246731
Ddr1	-3.31	0.04959382	0.388431941
Adamts13	-1.973333333	0.049677947	0.388744549
Fam149b1	-8.033333333	0.049686761	0.388744549
Itgad	-5.82	0.049804324	0.388956352
Kcna3	-2.526666667	0.049839713	0.388956352
Cyyr1	-3.343333333	0.049840675	0.388956352
Senp7	-1.64	0.049904466	0.388956352
Ube2l6	-5.88	0.04995289	0.388956352
Angptl8	-1.076666667	0.049953705	0.388956352
Prss35	-3.416666667	0.049973535	0.388956352
Acp2	-1.736666667	0.049979941	0.388956352
